# Impact of female underrepresentation in trials investigating long-term pharmacologic therapy after acute coronary syndrome: a meta-analysis and meta-regression

**DOI:** 10.1093/ehjacc/zuag020

**Published:** 2026-02-04

**Authors:** Marte F van der Bijl, Lotte A Paulis, Hester M den Ruijter, Iris C D Westendorp, Astrid Schut, Yolande Appelman, Jeanine E Roeters van Lennep, Eric Boersma

**Affiliations:** Department of Internal Medicine, Erasmus MC, University Medical Center, Rotterdam, The Netherlands; Department of Internal Medicine, Erasmus MC, University Medical Center, Rotterdam, The Netherlands; Laboratory for Experimental Cardiology, Department of Cardiology, University Medical Center Utrecht, Utrecht University, Utrecht, The Netherlands; Department of Cardiology, Red Cross Hospital, Beverwijk, The Netherlands; WCN (Dutch Network for Cardiovascular Research), Utrecht, The Netherlands; WCN (Dutch Network for Cardiovascular Research), Utrecht, The Netherlands; Department of Cardiology, Amsterdam Heart Centre, Amsterdam Cardiovascular Sciences, VU University Medical Center, Amsterdam, The Netherlands; Department of Internal Medicine, Erasmus MC, University Medical Center, Rotterdam, The Netherlands; Department of Cardiology, Cardiovascular Institute, Erasmus MC, University Medical Center, PO Box 2040, Rotterdam CA 3000, The Netherlands

**Keywords:** Trials, Sex differences, Meta-analysis, Acute coronary syndrome

## Abstract

**Aims:**

Female underrepresentation in clinical trials of acute coronary syndromes (ACS) may hinder the assessment of sex-based differences in the outcomes of long-term pharmacological therapy. The presence of these differences and their potential association with female representation in clinical trials remain unclear.

**Methods and results:**

A systematic search of Embase, Medline Ovid, and Cochrane Central was conducted through 1 July 2025, in accordance with the reporting standards of the Preferred Reporting Items for Systematic Reviews and Meta-Analyses guidelines. Eligible randomized controlled trials (RCTs) compared long-term pharmacological therapy for ACS with placebo or standard care, included ≥1-year follow-up, and reported a clinical event as the primary outcome. Sex differences in treatment effects were analysed using a random-effects meta-analysis, while meta-regression was used to assess the association between the proportion of females in each trial and these differences. The main outcome was the sex difference in the relative effect measure (REM; mostly a hazard ratio) for the primary efficacy endpoint. Among 102 RCTs, female representation ranged from 10 to 52%. Forty-eight trials provided sex-stratified data. Pooled analysis showed no evidence of sex-related differences in efficacy: the mean difference in the log of the REM of males minus females was 0.00 (95% confidence interval, −0.05–0.05; *P* = 0.98; heterogeneity *I*² = 0%). Meta-regression indicated no relationship between female trial participation and sex-specific treatment effects.

**Conclusion:**

In RCTs of long-term pharmacological therapy after ACS, treatment efficacy was comparable between sexes, irrespective of sex distribution. These findings support current guidelines recommending equivalent long-term pharmacological strategies for secondary prevention in both sexes.

## Graphical Abstract

**Graphical Abstract:**
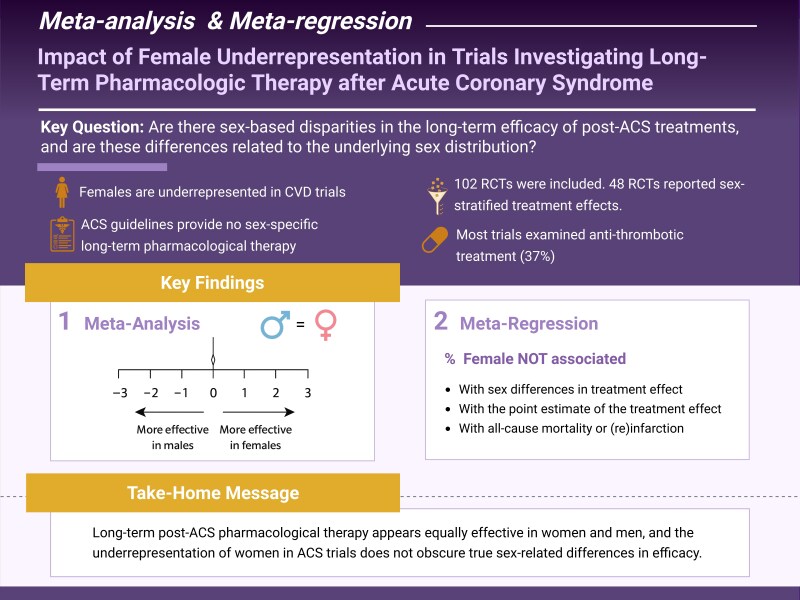

## Introduction

Cardiovascular disease (CVD) represents the leading cause of death among females worldwide.^1^ In Europe, analyses accounting for ischaemic heart disease mortality relative to disease prevalence indicate that females have higher age-standardized mortality rates than males across most countries. This suggests that, despite lower overall disease prevalence, females face a greater risk of death within the same age group.^2^ According to the 2025 American College of Cardiology/American Heart Association guidelines for the management of acute coronary syndrome (ACS), as well as the 2023 European Society of Cardiology guidelines, similar long-term pharmacological treatment is recommended to reduce morbidity and mortality in females and males.^3,4^ The guidelines underscore the need for further research into the role of personalized medicine in the long-term management of ACS and on the generalizability of current randomized controlled trial (RCT) findings, particularly for females, who remain underrepresented in current post-ACS trials.^4^

Given the established sex differences in body composition, pharmacokinetics, and pharmacodynamics, it is relevant to explore differential cardiovascular drug responses in females and males.^5^ However, sex differences in treatment effects are challenging to investigate due to the female/male imbalance in clinical trials. Female patients are underrepresented in CVD trials relative to the population prevalence.^6–8^ This underrepresentation may limit the generalizability of findings and may hinder accurate estimation of treatment effects in females.^9,10^ Moreover, individual trials are not adequately powered to detect differences within specific subgroups.^11^ Therefore, meta-research is recommended to elucidate possible heterogeneous treatment effects across sexes.^11^

Against this background, we studied sex disparities in the efficacy of long-term pharmacological therapies after ACS and their relation to trial sex distribution through a systematic analysis published RCTs.

## Methods

We performed a systematic review, meta-analysis and meta-regression of RCTs and complied with the reporting standards of the Preferred Reporting Items for Systematic Reviews and Meta-Analyses (PRISMA) guidelines.^12^ The PRISMA checklist can be found in Supplementary material online, Material  *S1*. This review is registered in the International Prospective Register of Systematic Reviews under the number CRD42024544347.

### Terminology

Sex was defined as biological sex, reflecting physiological and anatomical characteristics.^13^ Accordingly, the terms ‘female(s)’ and ‘male(s)’ are used throughout. We acknowledge the influence of gender-related factors on cardiovascular health and the limitations of binary classification, but present sex as documented in the original RCTs.

### Data sources

In collaboration with an information specialist of the medical library of our hospital, a literature search using Embase, Medline Ovid, and Cochrane Central Register of Controlled Trials was performed. The search included studies that were reported up until 1 July 2025. The search terms included ‘Acute Coronary Syndrome’, ‘Unstable angina pectoris’, ‘Myocardial infarction’, ‘Drug therapy’, and ‘Randomized controlled trial’. A detailed list of search terms is available in Supplementary material online, Material  *S2*.

### Study selection

Only published peer-reviewed original articles in English were included. Trials were eligible for inclusion if they were RCTs, comparing any long-term pharmacological treatment (≥1 year) for ACS with placebo or standard care in patients aged ≥18 years. Patients needed to have experienced an ACS within 3 months before enrolment. To minimize the likelihood of small-study effects, all trials were required to have at least 100 participants.^14^ The primary outcome was required to be a clinical efficacy event as defined by the trial, with a minimum protocol-defined follow-up of 1 year. Acute coronary syndrome encompassed unstable angina and acute myocardial infarction (MI) with or without ST-elevation. Given the evolving definition of ACS, older classifications such as (non-)Q wave MI and (non-)transmural MI were also considered within the ACS diagnosis. Studies that conducted analyses of previously investigated populations were excluded. However, if a secondary analysis identified during the search suggested that a relevant original study had not been captured, the original article was subsequently retrieved and included. Full eligibility criteria are presented in *Table 1*.

**Table 1:** In- and exclusion criteria

**Inclusion criteria patients:**
Patients with ACS (<3 months ago)
≥18 years
**Exclusion criteria patients:**
Non-ACS patient population
**Inclusion criteria studies:**
RCT with a sample size of ≥100 patients
English language
Full text available
Pharmacological intervention for ≥1 year Primary event-driven outcome Reports % male vs. female in study population
**Exclusion criteria studies:**
Design papers, preliminary reports, conference papers, post-trial follow-ups, (systematic) reviews, and meta-analyses Not original study Non-pharmaceutical intervention Follow-up schedules ≤ 1 year

ACS, acute coronary syndrome.

Two co-authors (M.v.d.B. and L.P.) independently screened title and abstract to identify articles for inclusion, with disagreements resolved by a third co-author (E.B.). Screening was performed using Covidence.^15^ Full texts of the selected studies were then independently reviewed by the same authors, with eligibility assessed according to predefined inclusion and exclusion criteria; disagreements were again resolved by E.B.

### Data extraction and quality assessment

Two co-authors (M..v.d.B. and L.P.) independently extracted data from studies that met inclusion criteria using a standardized extraction form. The following data was systematically collected: details of interventions (e.g. type of medication and dosage), patient characteristics (e.g. age, sex, and diagnosis), study details (e.g. publication year and trial name), the relative and absolute measures for the primary event-driven outcome (if available stratified per sex), and the absolute numbers for all-cause mortality, re-infarction, and stroke. Following extraction, the results were checked for consistency, and any discrepancies were solved by reconsulting the original study or, if needed, through discussion with E.B. Trials with a factorial design, in which multiple treatments were compared, had all treatment arms extracted and included in the analysis. When the primary outcome was not stratified by sex in the original publication, secondary sex-stratified analyses were sought. Inclusion and exclusion criteria were reviewed for all included RCTs, using either the original publication or associated rationale article, to assess whether any criteria explicitly differed for females and males.

Two co-authors (M.v.d.B. and L.P.) independently appraised the potential risks of bias of the randomized clinical trials using the Cochrane Risk of Bias Tool.^16^

### Data synthesis and analysis

Trial characteristics are presented as means with ranges. Patient characteristics are presented as weighted means with ranges for continues variables and mean percentage with range for categorical variables.

Within the meta-analysis, we used the sex-stratified, natural log-transformed relative effect measure (REM) of the primary efficacy outcome defined by each trial. Because the primary outcomes differed across trials, the key endpoint of our analyses was the difference in log(REM) between females and males. For clarity, a delta log(REM) of zero indicates no difference in treatment effect between females and males. Random-effects methods, using the DerSimonian and Laird procedure, were applied to estimate the pooled delta log(REM), which is reported with its corresponding 95% confidence interval (CI).

The different pharmacological interventions tested in the trials were further categorized based on treatment entities used after ACS: antithrombotic therapy, lipid-lowering therapy, beta-blockers, and Renin-Angiotensin-Aldosterone System (RAAS) inhibition, including angiotensin-converting enzyme inhibitors and angiotensin receptor blockers.^3,4^ Interventions that did not fit into these categories were classified as ‘other’. Heterogeneity was assessed using the I^2^ statistic and classified as not important (*I*², ≤25%), moderate, (*I*², 26–50%), substantial (*I*², 51–75%), and considerable (*I*², >75%).^17^ Funnel plots were generated and Egger regression tests performed to assess publication bias.^18^ Sensitivity analyses were conducted to evaluate the impact of including only placebo-controlled and low risk of bias studies.

A meta-regression was conducted to examine the relationship between the proportion of females in each trial and the delta log[REM] between females and males (our key endpoint), as well as with log[REM], the overall efficacy estimate. Additionally, meta-regression was performed to examine the relationship between the percentage of female participants and the log[Odds Ratio (OR)] for all-cause-mortality, MI, and stroke, if available.

All analyses and visualizations were performed using the ‘meta’ and ‘metafor’ packages in R Studio (version 4.2.2). A two-sided *P*-value of ≤0.05 was considered as statistically significant for all comparisons.

## Results

The literature search yielded 7315 studies after removing duplicates. Of these, 100 trials met all eligibility criteria and were included in the analysis. Additionally, two original trials were identified from non-original articles that seemed relevant during full-text screening, bringing the total to 102 trials. The PRISMA flow diagram is presented in Supplementary material online, *Figure S1*.

The trials collectively included 309 009 ACS patients. The percentage of female participants ranged from 10 to 52%. Due to the premature discontinuation of eight trials, three of which had a mean or median follow-up of <1 year, the follow-up period varied from 225 days to 6 years. The included trials examined a range of primary efficacy endpoints and pharmacological interventions. Most included trials were examining anti-thrombotic treatment (37% of all included trials). Primary endpoints varied, ranging from all-cause mortality to composite cardiovascular outcomes (in 75% of trials), most often including MI and stroke. In 49% of trials, different in- and exclusion criteria were applied for females and males. Most commonly, females were excluded only if pregnant or breastfeeding, whereas participation was permitted with the use of adequate contraception. The trial and patient characteristics of the included studies are displayed in Supplementary material online, *Table S1*.

### Sex differences in efficacy of pharmacological treatment of acute coronary syndrome

Sex-stratified information on the REM of the primary outcome was available in 48 RCTs (see Supplementary material online, *Table S2*), including three RCTs with multiple treatment arms. These trials included 249 729 patients (81% of the included patients), 62 779 of whom were females (82% of the included females). The largest proportion of trials were categorized as investigating antithrombotic treatment (44% of all trials). Among the trials included in the meta-analysis, 71% reported sex-specific inclusion and exclusion criteria, most commonly excluding pregnant or breastfeeding females and/or requiring agreement to use contraception.

**Figure 1:**
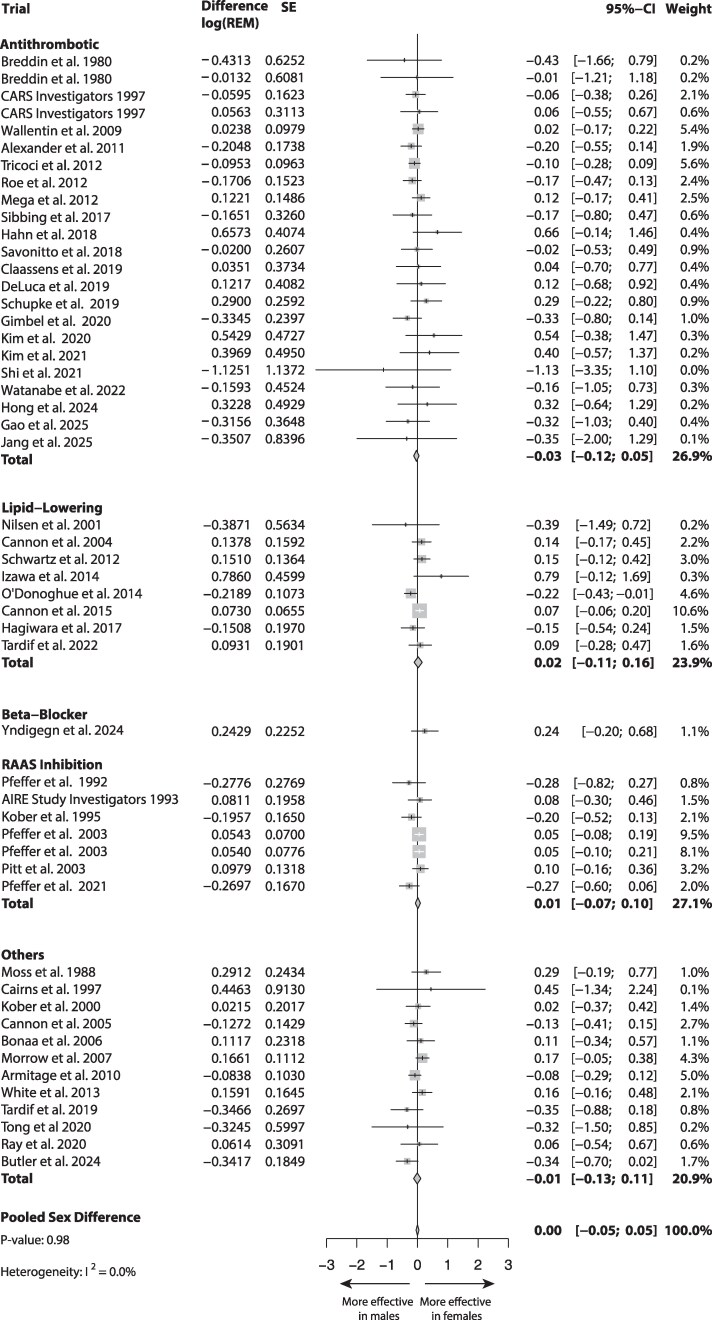
Forest plot of sex differences in the relative effect measure from the meta-analysis.

The pooled analysis showed no sex differences in the primary outcome between female and male ACS patients on long-term pharmacological therapy (delta log[REM], 0.00; 95% CI, −0.05–0.05; *P*-value, 0.98; heterogeneity *I*^2^, 0%; *Figure 1*]. No differences were observed across treatment domains either.

### Risk of bias and sensitivity analyses

Potential sources of bias are shown in Supplementary material online, *Figure S2*. Sensitivity analyses restricted to studies with low risk of bias (26 studies) and to placebo-controlled trials (23 studies) confirmed the overall meta-analysis findings. The visual inspection of the funnel plot and Egger’s regression test showed no evidence of publication bias (*P* = 0.68) (see Supplementary material online, *Figure S3*).

### Meta-regression

**Figure 2:**
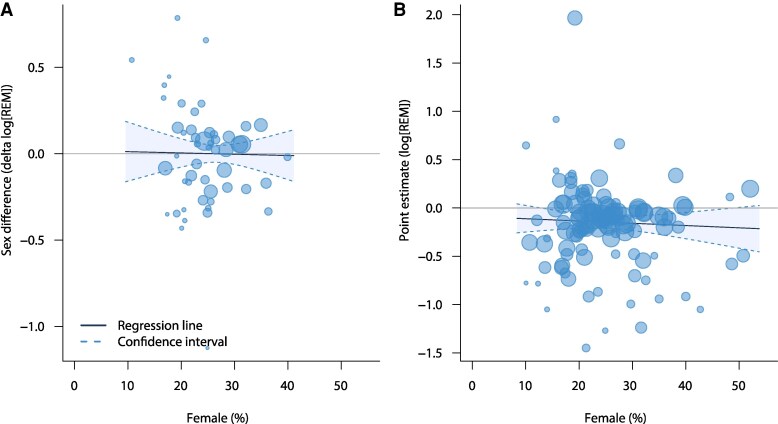
Bubble plot of the meta-regression analysing the proportion of female participants and (*A*) sex differences in the relative effect measure and (*B*) the point estimate of the relative effect measure.

The proportion of female participants in a trial was not associated with sex differences in treatment effect (delta log[REM]) (*Figure 2A*; regression slope, 0.00; 95% CI, −0.01–0.01; *P* = 0.88.). Similarly, based on all 102 trials, the proportion of female participants was not associated with the point estimate of the primary efficacy endpoint (log[REM]) (*Figure 2B*; regression slope, 0.00; 95% CI, −0.01–0.01; *P* = 0.57).

**Figure 3:**
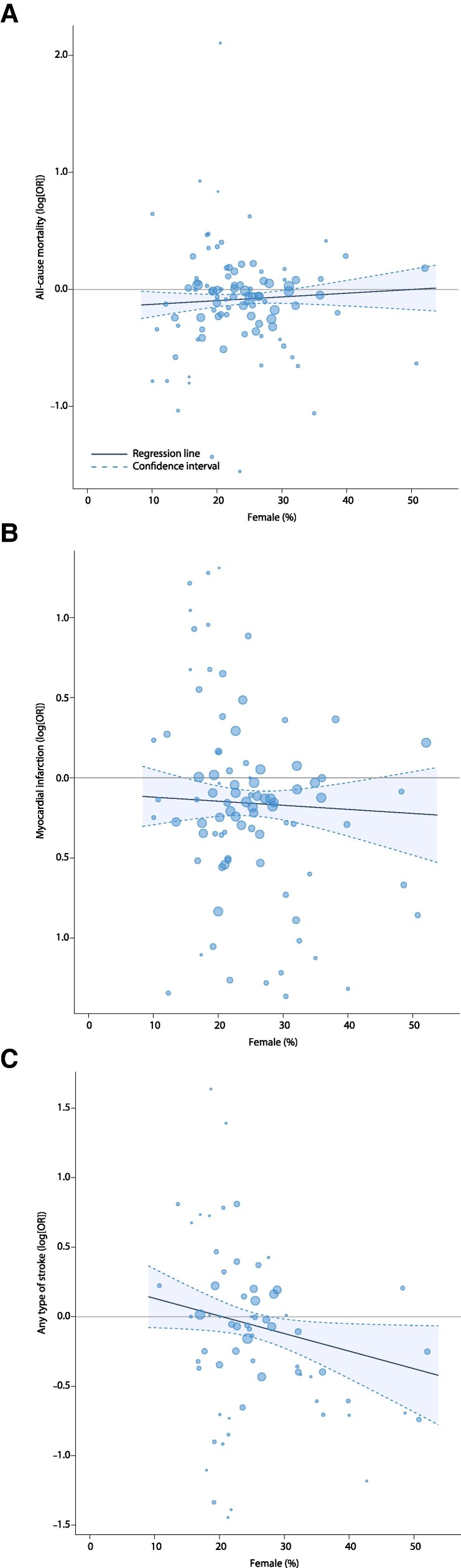
Bubble plots of the meta-regression examining the proportion of female participants and outcomes of (*A*) all-cause mortality, (*B*) myocardial infarction, and (*C*) stroke.

Furthermore, no association was found between female representation and the overall OR for all-cause mortality or (re-)infarction. However, a higher proportion of female participants was significantly associated with a lower OR for any type of stroke during follow-up (regression slope, −0.01; 95% CI, −0.02–0.00; *P* = 0.04). Specifically, for every 10% increase in the proportion of female participants, the OR for stroke in favour of the experimental pharmacological treatment decreased by a factor of 0.12, suggesting that females had better stroke protection than males. These findings are presented in *Figure 3A–C*.

## Discussion

Our meta-analysis and meta-regression indicate that a difference in the efficacy of long-term pharmacologic therapy between females and males with ACS is unlikely. Not only was the pooled estimate virtually equal to zero, but the 95% CI was also narrow, corresponding to a maximum difference of about 5% in relative effect in either direction [for reference, exp(0.05) ∼ 1.05]. Furthermore, the proportion of female participants did not affect point estimates of treatment effect on the primary outcome, all-cause mortality, and MI. The only exception was stroke, with possibly a somewhat greater treatment effect in females.

Our findings largely align with a meta-analysis assessing the efficacy of potent P2Y12 inhibitors in 63 346 males and 24 494 females with coronary artery disease (CAD), which found no significant difference in efficacy between sexes.^19^ Similarly, a previous meta-analysis of LDL-lowering therapy involving 174 000 patients with 46 675 females demonstrated that statin therapy was comparably effective in preventing major vascular events in both females and males.^20^ Noteworthy, our analysis extends beyond a single drug class by encompassing all long-term therapies administered post-ACS in any research setting and focusing on the potential impact of female underrepresentation in ACS trials.

We found some evidence suggesting a stronger protective effect of long-term pharmacologic therapy on stroke in females than in males, for which no clear explanation emerged in the available data. Importantly, no sex-specific differences appeared with regard to composite endpoints that include stroke. Furthermore, the association was weakened and no longer significant in a meta-regression of the low risk of bias trials (regression slope, −0.02; 95% CI, −0.04–0.00; *P* = 0.12). If any difference were to exist, it would most likely be attributable to variations in trial design, baseline characteristics, or the specific effect of the drug on stroke compared with other cardiovascular endpoints. For now, we consider this a coincidental finding, albeit one that warrants further investigation. Such investigation would require access to individual patient data.

In the ongoing debate regarding the female underrepresentation in CVD trials, CAD continues to demonstrate significant underrepresentation.^21,22^ This raises the concern that potential sex differences in treatment effects may go undetected, leading to suboptimal care for females.^9,10^ However, RCTs are typically powered to detect overall homogeneous effects, not subgroup differences, and sex-specific analyses often lack sufficient power.^11^ While sex-stratified reporting is a necessary step forward, results must be interpreted cautiously in the absence of prespecified hypotheses and adequate power as any observed differences may be attributable to chance. To address this limitation, we found in our pooled analysis no evidence of systematic sex differences in treatment efficacy, either overall or by medication class. The narrow CIs and low heterogeneity suggest a low likelihood of meaningful sex differences being missed. These results support the applicability of current clinical guidelines, which primarily derive their recommendations from RCTs with clearly defined primary outcomes. Nonetheless, it is important that composite endpoints consider sex differences in specific outcomes, such as stroke, as the inclusion or exclusion of such outcomes may impact overall sex-specific effects.^23^

In clinical practice, treatment decisions are guided by both efficacy and safety, particularly adverse drug reactions (ADRs). Global data show that ADRs are reported more frequently by females.^24^ Pharmacokinetic studies have found that females often have higher drug concentrations and longer drug elimination times for many Food and Drug Administration-approved drugs, contributing to increased ADR incidence.^25^ Despite this, females remain underrepresented in early-phase trials where dosing is established,^26^ and safety data are often not reported in a sex-stratified way. Given our findings of no overall sex difference in treatment efficacy, while existing literature points to a higher ADR incidence, we propose shifting the focus beyond equity in Phase III RCTs (most often focusing on efficacy) towards evaluating sex-specific differences in dosing and safety. This requires adequately powered, sex-balanced Phase II dose-finding trials, routine sex-stratified reporting of ADRs in trials, and comprehensive safety registries to capture sex-specific side-effect profiles.

### Strengths and limitations

To the best of our knowledge, this meta-analysis is the first comprehensive evaluation of sex differences in the efficacy of long-term pharmaceutical treatments for ACS patients. A key innovation of our study is the use of meta-regression analysis to assess whether sex differences in treatment efficacy are influenced by the proportion of female participants in the included trials. Additionally, the large sample size, inclusion of trials from diverse geographical regions, and consideration of all-ever tested long-term pharmaceutical treatments—spanning a broad timeframe from 1971 to 2025—enhance the generalizability of our findings and strengthen the robustness of our conclusions. The low heterogeneity observed in the primary analyses and the narrow CIs support the reliability of these findings, suggesting that sex differences in efficacy of the primary outcome were unlikely to have been overlooked.

However, several limitations should be acknowledged. First, this meta-analysis focuses solely on pharmaceutical therapies for ACS, excluding non-pharmaceutical interventions, such as stents and cardiac devices, which are also integral to ACS management. Second, we focused exclusively on long-term pharmaceutical therapies, excluding acute treatments to maintain methodological consistency. Additionally, phenotypes that are more pronounced in females, such as spontaneous coronary artery dissection, were not considered in this analysis, as large-scale trials on this topic have not been conducted.

Third, only 48 of the 102 identified trials were included in the primary analysis, as the remaining trials did not report sex-specific estimates on the primary endpoint. Importantly, these trials represent the majority (82%) of females across all studies. Nevertheless, the exclusion of trials without sex-specific reporting may have introduced selection bias if reporting trials differed systematically from nonreporting studies. Although this issue should be noted, the reporting of sex-specific data has significantly improved in recent years, with any potential bias likely stemming from the older trials. Importantly, the present analysis includes all major categories of long-term pharmacological treatment used in clinical practice, namely, antithrombotic therapy, RAAS inhibition, lipid-lowering treatment, and beta-blockers. The included trials assessed a range of endpoints. Although this variability does not compromise internal validity, as only (differences in) REMs were used, it may, to some extent, limit the external validity of the findings. Moreover, there is limited availability of sex-specific data for individual outcomes, such as all-cause mortality and MI, which are often secondary endpoints but rarely reported in a sex-stratified manner. Finally, this meta-analysis used aggregated data. A meta-analysis of individual participant data from these trials could provide more detailed insights into sex-specific baseline characteristics and their potential influence on treatment effects.

## Conclusions

In this comprehensive meta-analysis and meta-regression, no significant sex differences were observed in the efficacy of long-term pharmaceutical therapies for ACS. These findings provide reassurance that current treatments are equally effective in females and males. Given ongoing concerns about the underrepresentation of females in cardiovascular trials, our results suggest that differences in efficacy are unlikely to have been overlooked. Future research should prioritize sex-specific differences in drug safety and dosing, with a focus on more inclusive early-phase trials and registry-based studies to optimize treatment strategies for all patients.

## Supplementary Material

zuag020_Supplementary_Data

## Data Availability

Data will be provided upon reasonable request.

## References

[1] Vogel B, Acevedo M, Appelman Y, Bairey Merz CN, Chieffo A, Figtree GA, et al The lancet women and cardiovascular disease commission: reducing the global burden by 2030. Lancet 2021;397:2385–2438. 34010613 10.1016/S0140-6736(21)00684-X

[2] Maas A, Cenko E, Vaccarino V, Göttgens I, Bergami M, Manfrini O, et al Changing clinical perspectives on sex and healthcare disparities in ischaemic heart disease. Lancet Reg Health Eur 2025:56:101370. 41200015 10.1016/j.lanepe.2025.101370 PMC12587326

[3] Rao SV, O’Donoghue ML, Ruel M, Rab T, Tamis-Holland JE, Alexander JH, et al 2025 ACC/AHA/ACEP/NAEMSP/SCAI guideline for the management of patients with acute coronary syndromes: a report of the American College of Cardiology/American Heart Association joint committee on clinical practice guidelines. Circulation 2025;151:e771–e862. 40014670 10.1161/CIR.0000000000001309

[4] Byrne RA, Rossello X, Coughlan JJ, Barbato E, Berry C, Chieffo A, et al 2023 ESC guidelines for the management of acute coronary syndromes. Eur Heart J Acute Cardiovasc Care 2024;13:55–161. 37740496 10.1093/ehjacc/zuad107

[5] Tamargo J, Rosano G, Walther T, Duarte J, Niessner A, Kaski JC, et al Gender differences in the effects of cardiovascular drugs. Eur Heart J Cardiovasc Pharmacother 2017;3:163–182. 28329228 10.1093/ehjcvp/pvw042

[6] van der Bijl MF, Roeters van Lennep JE, Schut A, Westendorp ICD, Appelman Y, den Ruijter HM, et al A comprehensive analysis of female participation in cardiovascular trials involving the WCN investigator network. Neth Heart J 2025;33:404–411. 41222889 10.1007/s12471-025-01999-4 PMC12638513

[7] Rivera FB, Magalong JV, Bantayan NRB, Tesoro N, Milan MJ, Purewal V, et al Participation of women in cardiovascular trials from 2017 to 2023: a systematic review. JAMA Netw Open 2025;8:e2529104. 40886088 10.1001/jamanetworkopen.2025.29104 PMC12400126

[8] Matthews S, Cook S, Clayton T, Murray S, Wynne R, Sanders J. Factors affecting women's participation in cardiovascular research: a scoping review. Eur J Cardiovasc Nurs 2024;23:107–114. 37201192 10.1093/eurjcn/zvad048

[9] Filbey L, Khan MS, Van Spall HGC. Protection by inclusion: increasing enrollment of women in cardiovascular trials. Am Heart J Plus 2022;13:100091. 38560056 10.1016/j.ahjo.2022.100091 PMC10978184

[10] Filbey L, Zhu JW, D'Angelo F, Thabane L, Khan MS, Lewis E, et al Improving representativeness in trials: a call to action from the global cardiovascular clinical trialists forum. Eur Heart J 2023;44:921–930. 36702610 10.1093/eurheartj/ehac810 PMC10226751

[11] Aaron LS, Marcella A, Alanna AM, Scott DH. Why diverse clinical trial participation matters. N Engl J Med 2023;388:1252–1254. 37017480 10.1056/NEJMp2215609

[12] Page MJ, McKenzie JE, Bossuyt PM, Boutron I, Hoffmann TC, Mulrow CD, et al The PRISMA 2020 statement: an updated guideline for reporting systematic reviews. Bmj 2021;372:n71. 33782057 10.1136/bmj.n71 PMC8005924

[13] Canadian Institutes of Health Research. What is gender? What is Sex? 2023 [cited 2025 29th of July]. Available from: https://cihr-irsc.gc.ca/e/48642.html.

[14] Kjaergard LL, Villumsen J, Gluud C. Reported methodologic quality and discrepancies between large and small randomized trials in meta-analyses. Ann Intern Med 2001;135:982–989. 11730399 10.7326/0003-4819-135-11-200112040-00010

[15] Covidence Systematic Review Software. Melbourne, Australia: Veritas Health Innovation. www.covidence.org.

[16] Sterne JAC, Savović J, Page MJ, Elbers RG, Blencowe NS, Boutron I, et al Rob 2: a revised tool for assessing risk of bias in randomised trials. Bmj 2019;366:l4898. 31462531 10.1136/bmj.l4898

[17] Higgins JP, Thompson SG, Deeks JJ, Altman DG. Measuring inconsistency in meta-analyses. Bmj 2003;327:557–560. 12958120 10.1136/bmj.327.7414.557 PMC192859

[18] Egger M, Davey Smith G, Schneider M, Minder C. Bias in meta-analysis detected by a simple, graphical test. Bmj 1997;315:629–634. 9310563 10.1136/bmj.315.7109.629 PMC2127453

[19] Lau ES, Braunwald E, Murphy SA, Wiviott SD, Bonaca MP, Husted S, et al Potent P2Y(12) inhibitors in men versus women: a collaborative meta-analysis of randomized trials. J Am Coll Cardiol 2017;69:1549–1559. 28335837 10.1016/j.jacc.2017.01.028

[20] Cholesterol Treatment Trialists C, Fulcher J, O'Connell R, Voysey M, Emberson J, Blackwell L, et al Efficacy and safety of LDL-lowering therapy among men and women: meta-analysis of individual data from 174,000 participants in 27 randomised trials. Lancet 2015;385:1397–1405. 25579834 10.1016/S0140-6736(14)61368-4

[21] Scott PE, Unger EF, Jenkins MR, Southworth MR, McDowell TY, Geller RJ, et al Participation of women in clinical trials supporting FDA approval of cardiovascular drugs. J Am Coll Cardiol 2018;71:1960–1969. 29724348 10.1016/j.jacc.2018.02.070

[22] Jin X, Chandramouli C, Allocco B, Gong E, Lam CSP, Yan LL. Women's participation in cardiovascular clinical trials from 2010 to 2017. Circulation 2020;141:540–548. 32065763 10.1161/CIRCULATIONAHA.119.043594

[23] Gómez G, Gómez-Mateu M, Dafni U. Informed choice of composite End points in cardiovascular trials. Circ Cardiovasc Qual Outcomes 2014;7:170–178. 24425702 10.1161/CIRCOUTCOMES.113.000149

[24] Watson S, Caster O, Rochon PA, den Ruijter H. Reported adverse drug reactions in women and men: aggregated evidence from globally collected individual case reports during half a century. EClinicalMedicine 2019;17:100188. 31891132 10.1016/j.eclinm.2019.10.001 PMC6933269

[25] Zucker I, Prendergast BJ. Sex differences in pharmacokinetics predict adverse drug reactions in women. Biol Sex Differ 2020;11:32. 32503637 10.1186/s13293-020-00308-5 PMC7275616

[26] Sosinsky AZ, Rich-Edwards JW, Wiley A, Wright K, Spagnolo PA, Joffe H. Enrollment of female participants in United States drug and device phase 1–3 clinical trials between 2016 and 2019. Contemp Clin Trials 2022;115:106718. 35247632 10.1016/j.cct.2022.106718

