# Supplementary material for: Impact of female underrepresentation in trials investigating long-term pharmacologic therapy after acute coronary syndrome: a meta-analysis and meta-regression
Source: Eur Heart J Acute Cardiovasc Care. 2026 Feb 4;15(5):375–82. doi: 10.1093/ehjacc/zuag020 (PMC13185942; doi:10.1093/ehjacc/zuag020)
Supplement: zuag020_Supplementary_Data [file zuag020_supplementary_data.docx]

Supplemental Documents

[**Supplemental Material 1**. PRISMA checklist 2](#_Toc216791493)

[**Supplemental Material 2**. Search Strategy 5](#_Toc216791494)

[**Supplemental Figure 1**. Search Results and PRISMA flowchart 9](#_Toc216791495)

[**Supplemental Figure 2.** Risk of bias summary 10](#_Toc216791496)

[**Supplemental Figure 3**. Funnel plot of the meta-analysis of sex differences 11](#_Toc216791497)

[**Supplemental Table 1.** Trial and patient characteristics of all included trials (1-102) 22](#_Toc216791498)

[**Supplemental Table 2**. Sex -specific relative effect measures of the primary endpoint 29](#_Toc216791499)

# **Supplemental Material 1**. PRISMA checklist

| **Section and Topic** | **Item #** | **Checklist item** | **Location where item is reported** |
| --- | --- | --- | --- |
| **TITLE** | | |  |
| Title | 1 | Identify the report as a systematic review. | 1 |
| **ABSTRACT** | | |  |
| Abstract | 2 | See the PRISMA 2020 for Abstracts checklist. | 3 |
| **INTRODUCTION** | | |  |
| Rationale | 3 | Describe the rationale for the review in the context of existing knowledge. | 4 |
| Objectives | 4 | Provide an explicit statement of the objective(s) or question(s) the review addresses. | 4 |
| **METHODS** | | |  |
| Eligibility criteria | 5 | Specify the inclusion and exclusion criteria for the review and how studies were grouped for the syntheses. | 5 and Supplemental Material 1. |
| Information sources | 6 | Specify all databases, registers, websites, organisations, reference lists and other sources searched or consulted to identify studies. Specify the date when each source was last searched or consulted. | 5 and Supplemental Material 1. |
| Search strategy | 7 | Present the full search strategies for all databases, registers and websites, including any filters and limits used. | Supplemental Material 1 |
| Selection process | 8 | Specify the methods used to decide whether a study met the inclusion criteria of the review, including how many reviewers screened each record and each report retrieved, whether they worked independently, and if applicable, details of automation tools used in the process. | 5 and Supplemental Material 1. |
| Data collection process | 9 | Specify the methods used to collect data from reports, including how many reviewers collected data from each report, whether they worked independently, any processes for obtaining or confirming data from study investigators, and if applicable, details of automation tools used in the process. | 6 |
| Data items | 10a | List and define all outcomes for which data were sought. Specify whether all results that were compatible with each outcome domain in each study were sought (e.g. for all measures, time points, analyses), and if not, the methods used to decide which results to collect. | 6 |
|  | 10b | List and define all other variables for which data were sought (e.g. participant and intervention characteristics, funding sources). Describe any assumptions made about any missing or unclear information. | 6 |
| Study risk of bias assessment | 11 | Specify the methods used to assess risk of bias in the included studies, including details of the tool(s) used, how many reviewers assessed each study and whether they worked independently, and if applicable, details of automation tools used in the process. | 6 |
| Effect measures | 12 | Specify for each outcome the effect measure(s) (e.g. risk ratio, mean difference) used in the synthesis or presentation of results. | 6 |
| Synthesis methods | 13a | Describe the processes used to decide which studies were eligible for each synthesis (e.g. tabulating the study intervention characteristics and comparing against the planned groups for each synthesis (item #5)). | 6 |
|  | 13b | Describe any methods required to prepare the data for presentation or synthesis, such as handling of missing summary statistics, or data conversions. | 6 |
|  | 13c | Describe any methods used to tabulate or visually display results of individual studies and syntheses. | 7 |
|  | 13d | Describe any methods used to synthesize results and provide a rationale for the choice(s). If meta-analysis was performed, describe the model(s), method(s) to identify the presence and extent of statistical heterogeneity, and software package(s) used. | 6 |
|  | 13e | Describe any methods used to explore possible causes of heterogeneity among study results (e.g. subgroup analysis, meta-regression). | 6-7 |
|  | 13f | Describe any sensitivity analyses conducted to assess robustness of the synthesized results. | 6 |
| Reporting bias assessment | 14 | Describe any methods used to assess risk of bias due to missing results in a synthesis (arising from reporting biases). | 6 |
| Certainty assessment | 15 | Describe any methods used to assess certainty (or confidence) in the body of evidence for an outcome. | - |
| **RESULTS** | | |  |
| Study selection | 16a | Describe the results of the search and selection process, from the number of records identified in the search to the number of studies included in the review, ideally using a flow diagram. | 8 |
|  | 16b | Cite studies that might appear to meet the inclusion criteria, but which were excluded, and explain why they were excluded. | Supplemental Figure 1 |
| Study characteristics | 17 | Cite each included study and present its characteristics. | 8 and Supplementary Table 1 |
| Risk of bias in studies | 18 | Present assessments of risk of bias for each included study. | 8-9 Supplementary Figure 2 |
| Results of individual studies | 19 | For all outcomes, present, for each study: (a) summary statistics for each group (where appropriate) and (b) an effect estimate and its precision (e.g. confidence/credible interval), ideally using structured tables or plots. | 8 and Supplementary Table 1-2 |
| Results of syntheses | 20a | For each synthesis, briefly summarise the characteristics and risk of bias among contributing studies. | 8 |
|  | 20b | Present results of all statistical syntheses conducted. If meta-analysis was done, present for each the summary estimate and its precision (e.g. confidence/credible interval) and measures of statistical heterogeneity. If comparing groups, describe the direction of the effect. | 8 |
|  | 20c | Present results of all investigations of possible causes of heterogeneity among study results. | 8 |
|  | 20d | Present results of all sensitivity analyses conducted to assess the robustness of the synthesized results. | 8-9 |
| Reporting biases | 21 | Present assessments of risk of bias due to missing results (arising from reporting biases) for each synthesis assessed. | 8-9 |
| Certainty of evidence | 22 | Present assessments of certainty (or confidence) in the body of evidence for each outcome assessed. | - |
| **DISCUSSION** | | |  |
| Discussion | 23a | Provide a general interpretation of the results in the context of other evidence. | 10-11 |
|  | 23b | Discuss any limitations of the evidence included in the review. | 11-12 |
|  | 23c | Discuss any limitations of the review processes used. | 11-12 |
|  | 23d | Discuss implications of the results for practice, policy, and future research. | 10-11 |
| **OTHER INFORMATION** | | |  |
| Registration and protocol | 24a | Provide registration information for the review, including register name and registration number, or state that the review was not registered. | 5 |
|  | 24b | Indicate where the review protocol can be accessed, or state that a protocol was not prepared. | 5 |
|  | 24c | Describe and explain any amendments to information provided at registration or in the protocol. | - |
| Support | 25 | Describe sources of financial or non-financial support for the review, and the role of the funders or sponsors in the review. | 13 |
| Competing interests | 26 | Declare any competing interests of review authors. | 13 |
| Availability of data, code and other materials | 27 | Report which of the following are publicly available and where they can be found: template data collection forms; data extracted from included studies; data used for all analyses; analytic code; any other materials used in the review. | Upon reasonable request: 13 |

*From:*  Page MJ, McKenzie JE, Bossuyt PM, Boutron I, Hoffmann TC, Mulrow CD, et al. The PRISMA 2020 statement: an updated guideline for reporting systematic reviews. BMJ 2021;372:n71. doi: 10.1136/bmj.n71. This work is licensed under CC BY 4.0. To view a copy of this license, visit <https://creativecommons.org/licenses/by/4.0/>

# **Supplemental Material 2**. Search Strategy

| **Database searched** | **Platform** | **Years of coverage** | **Records** | **Records after duplicates removed** |
| --- | --- | --- | --- | --- |
| Medline ALL | Ovid | 1946 - Present | 3495 | 3489 |
| Embase | Embase.com | 1971 - Present | 3802 | 1983 |
| Cochrane Central Register of Controlled Trials* | Wiley | 1992 - Present | 3880 | 1837 |
| **Total** | | | **11177** | **7309** |

* Manually deleted abstracts from trial registries

No other database limits were used than those specified in the search strategies

**Medline**

(* Acute Coronary Syndrome / OR * Angina, Unstable / OR exp * Myocardial Infarction / OR ((acute* ADJ3 coronar* ADJ3 syndrome*) OR (unstable ADJ3 angina*) OR ((heart OR myocard* OR cardiac*) ADJ3 (infarct* OR attack))).ti.) AND (exp Drug Therapy / OR * Acute Coronary Syndrome /dt OR * Angina, Unstable /dt OR exp * Myocardial Infarction /dt OR ((drug ADJ3 therap*)).ab,ti,kw.) AND (randomized controlled trial/ OR ((random* ADJ3 (stud* OR trial))).ab,ti,kw.) AND ((( 0## OR 1## OR 2## OR 3## OR 4## OR 5## OR 6## OR 7## OR 8## OR 9## OR 1### OR 2### OR 3### OR 4### OR 5### OR 6### OR 7### OR 8### OR 9###) ADJ6 (patients OR patient-record* OR patient-file* OR subjects OR individuals OR cases OR persons OR men OR women OR males OR females OR participant* OR people OR children OR adolescent* OR boys OR girls OR teens OR teenagers OR infants OR newborns OR elderly OR survivor* OR specimen* OR sample* OR episode* OR isolate* OR pediatric OR paediatric OR adult* OR surgeries OR operations OR observations OR mother* OR father* OR neonate* OR volunt* OR recruit* *OR infect* OR donor* OR presented* OR treatments)) OR ((hundred* OR thousand* OR million*) ADJ6 (patients OR patient-record* OR patient-file* OR subjects OR individuals OR cases OR persons OR men OR women OR males OR females OR participant* OR people OR children OR adolescent* OR boys OR girls OR teens OR teenagers OR infants OR newborns OR elderly OR survivor* OR specimen* OR sample* OR episode* OR isolate* OR pediatric OR paediatric OR adult* OR surgeries OR operations OR observations OR mother* OR father* OR neonate* OR volunt* OR recruit* *OR infect* OR donor* OR presented* OR treatments)) OR ((n OR included OR recruited OR randomized OR randomized OR assigned OR a-total-of OR group-of OR groups-of OR cohort-of OR cohorts-of) ADJ (0## OR 1## OR 2## OR 3## OR 4## OR 5## OR 6## OR 7## OR 8## OR 9## OR 1### OR 2### OR 3### OR 4### OR 5### OR 6### OR 7### OR 8### OR 9###)) OR large*-cohort* OR population-based OR multi-register* OR multiregister* OR regist*-based OR multicent* OR multi-cent* OR national-stud* OR national-regist* OR N#0## OR N#1## OR N#2## OR N#3## OR N#4## OR N#5## OR N#6## OR N#7## OR N#8## OR N#9## OR N#1### OR N#2### OR N#3### OR N#4### OR N#5### OR N#6### OR N#7### OR N#8### OR N#9###).ab,ti.NOT (Systematic Review / OR Meta-Analysis / OR (systematic-review* OR meta-analys*).ti.) NOT (news OR congres* OR abstract* OR book* OR chapter* OR dissertation abstract*).pt. NOT (exp animals/ NOT humans/) AND english.la.

**Embase**

('acute coronary syndrome'/mj/exp OR 'unstable angina pectoris'/mj/exp OR 'heart infarction'/mj/exp OR ((acute* NEAR/3 coronar* NEAR/3 syndrome*) OR (unstable NEAR/3 angina*) OR ((heart OR myocard* OR cardiac*) NEAR/3 (infarct* OR attack))):ti) AND ('drug therapy'/exp OR 'acute coronary syndrome'/mj/exp/dd_dt OR 'unstable angina pectoris'/mj/exp/dd_dt OR 'heart infarction'/mj/exp/dd_dt OR ((drug NEAR/3 therap*)):ab,ti,kw) AND ('randomized controlled trial'/exp OR ((random* NEAR/3 (stud* OR trial))):Ab,ti,kw) AND (((00? OR 01? OR 02? OR 03? OR 04? OR 05? OR 06? OR 07? OR 08? OR 09? OR 10? OR 11? OR 12? OR 13? OR 14? OR 15? OR 16? OR 17? OR 18? OR 19? OR 20? OR 21? OR 22? OR 23? OR 24? OR 25? OR 26? OR 27? OR 28? OR 29? OR 30? OR 31? OR 32? OR 33? OR 34? OR 35? OR 36? OR 37? OR 38? OR 39? OR 40? OR 41? OR 42? OR 43? OR 44? OR 45? OR 46? OR 47? OR 48? OR 49? OR 50? OR 51? OR 52? OR 53? OR 54? OR 55? OR 56? OR 57? OR 58? OR 59? OR 60? OR 61? OR 62? OR 63? OR 64? OR 65? OR 66? OR 67? OR 68? OR 69? OR 70? OR 71? OR 72? OR 73? OR 74? OR 75? OR 76? OR 77? OR 78? OR 79? OR 80? OR 81? OR 82? OR 83? OR 84? OR 85? OR 86? OR 87? OR 88? OR 89? OR 90? OR 91? OR 92? OR 93? OR 94? OR 95? OR 96? OR 97? OR 98? OR 99? OR 10?? OR 11?? OR 12?? OR 13?? OR 14?? OR 15?? OR 16?? OR 17?? OR 18?? OR 19?? OR 20?? OR 21?? OR 22?? OR 23?? OR 24?? OR 25?? OR 26?? OR 27?? OR 28?? OR 29?? OR 30?? OR 31?? OR 32?? OR 33?? OR 34?? OR 35?? OR 36?? OR 37?? OR 38?? OR 39?? OR 40?? OR 41?? OR 42?? OR 43?? OR 44?? OR 45?? OR 46?? OR 47?? OR 48?? OR 49?? OR 50?? OR 51?? OR 52?? OR 53?? OR 54?? OR 55?? OR 56?? OR 57?? OR 58?? OR 59?? OR 60?? OR 61?? OR 62?? OR 63?? OR 64?? OR 65?? OR 66?? OR 67?? OR 68?? OR 69?? OR 70?? OR 71?? OR 72?? OR 73?? OR 74?? OR 75?? OR 76?? OR 77?? OR 78?? OR 79?? OR 80?? OR 81?? OR 82?? OR 83?? OR 84?? OR 85?? OR 86?? OR 87?? OR 88?? OR 89?? OR 90?? OR 91?? OR 92?? OR 93?? OR 94?? OR 95?? OR 96?? OR 97?? OR 98?? OR 99??) NEXT/6 (patients OR patient-record* OR patient-file* OR subjects OR individuals OR cases OR persons OR men OR women OR males OR females OR participant* OR people OR children OR adolescent* OR boys OR girls OR teens OR teenagers OR infants OR newborns OR elderly OR survivor* OR specimen* OR sample* OR episode* OR isolate* OR pediatric OR paediatric OR adult* OR surgeries OR operations OR observations OR mother* OR father* OR neonate* OR volunt* OR recruit* OR infect* OR donor* OR presented OR treatments )) OR ((hundred OR thousand OR million) NEXT/5 (patients OR patient-record* OR patient-file* OR subjects OR individuals OR cases OR persons OR men OR women OR participants OR people OR children OR adolescent* OR boys OR girls OR teens OR teenagers OR infants OR newborns OR elderly OR survivor* OR specimen* OR sample* OR episode* OR isolate* OR pediatric OR paediatric OR adult* OR surgeries OR operations OR observations OR mother* OR father* OR neonate* OR volunt* OR recruit* OR infect* OR donor* OR presented OR treatments)) OR ((n OR included OR recruited OR randomized OR randomized OR assigned OR a-total-of OR group-of OR groups-of OR cohort-of OR cohorts-of) NEXT/2 (00? OR 01? OR 02? OR 03? OR 04? OR 05? OR 06? OR 07? OR 08? OR 09? OR 10? OR 11? OR 12? OR 13? OR 14? OR 15? OR 16? OR 17? OR 18? OR 19? OR 20? OR 21? OR 22? OR 23? OR 24? OR 25? OR 26? OR 27? OR 28? OR 29? OR 30? OR 31? OR 32? OR 33? OR 34? OR 35? OR 36? OR 37? OR 38? OR 39? OR 40? OR 41? OR 42? OR 43? OR 44? OR 45? OR 46? OR 47? OR 48? OR 49? OR 50? OR 51? OR 52? OR 53? OR 54? OR 55? OR 56? OR 57? OR 58? OR 59? OR 60? OR 61? OR 62? OR 63? OR 64? OR 65? OR 66? OR 67? OR 68? OR 69? OR 70? OR 71? OR 72? OR 73? OR 74? OR 75? OR 76? OR 77? OR 78? OR 79? OR 80? OR 81? OR 82? OR 83? OR 84? OR 85? OR 86? OR 87? OR 88? OR 89? OR 90? OR 91? OR 92? OR 93? OR 94? OR 95? OR 96? OR 97? OR 98? OR 99? OR 10?? OR 11?? OR 12?? OR 13?? OR 14?? OR 15?? OR 16?? OR 17?? OR 18?? OR 19?? OR 20?? OR 21?? OR 22?? OR 23?? OR 24?? OR 25?? OR 26?? OR 27?? OR 28?? OR 29?? OR 30?? OR 31?? OR 32?? OR 33?? OR 34?? OR 35?? OR 36?? OR 37?? OR 38?? OR 39?? OR 40?? OR 41?? OR 42?? OR 43?? OR 44?? OR 45?? OR 46?? OR 47?? OR 48?? OR 49?? OR 50?? OR 51?? OR 52?? OR 53?? OR 54?? OR 55?? OR 56?? OR 57?? OR 58?? OR 59?? OR 60?? OR 61?? OR 62?? OR 63?? OR 64?? OR 65?? OR 66?? OR 67?? OR 68?? OR 69?? OR 70?? OR 71?? OR 72?? OR 73?? OR 74?? OR 75?? OR 76?? OR 77?? OR 78?? OR 79?? OR 80?? OR 81?? OR 82?? OR 83?? OR 84?? OR 85?? OR 86?? OR 87?? OR 88?? OR 89?? OR 90?? OR 91?? OR 92?? OR 93?? OR 94?? OR 95?? OR 96?? OR 97?? OR 98?? OR 99??)) OR large*-cohort* OR population-based OR multi-register* OR multiregister* OR regist*-based OR multicent* OR multi-cent* OR national-stud* OR national-regist*):ab,ti NOT ('systematic review'/de OR 'meta analysis'/de OR (systematic-review* OR meta-analys*):ti) NOT [conference abstract]/lim NOT [editorial]/lim NOT [note]/lim NOT [letter]/lim NOT 'clinical trial'/it NOT ([animals]/lim NOT [humans]/lim) AND [english]/lim

**Cochrane**

(((acute* NEAR/3 coronar* NEAR/3 syndrome*) OR (unstable NEAR/3 angina*) OR ((heart OR myocard* OR cardiac*) NEAR/3 (infarct* OR attack))):ti) AND (((drug NEAR/3 therap*)):ab,ti,kw) AND (((random* NEAR/3 (stud* OR trial))):Ab,ti,kw) AND (((00? OR 01? OR 02? OR 03? OR 04? OR 05? OR 06? OR 07? OR 08? OR 09? OR 10? OR 11? OR 12? OR 13? OR 14? OR 15? OR 16? OR 17? OR 18? OR 19? OR 20? OR 21? OR 22? OR 23? OR 24? OR 25? OR 26? OR 27? OR 28? OR 29? OR 30? OR 31? OR 32? OR 33? OR 34? OR 35? OR 36? OR 37? OR 38? OR 39? OR 40? OR 41? OR 42? OR 43? OR 44? OR 45? OR 46? OR 47? OR 48? OR 49? OR 50? OR 51? OR 52? OR 53? OR 54? OR 55? OR 56? OR 57? OR 58? OR 59? OR 60? OR 61? OR 62? OR 63? OR 64? OR 65? OR 66? OR 67? OR 68? OR 69? OR 70? OR 71? OR 72? OR 73? OR 74? OR 75? OR 76? OR 77? OR 78? OR 79? OR 80? OR 81? OR 82? OR 83? OR 84? OR 85? OR 86? OR 87? OR 88? OR 89? OR 90? OR 91? OR 92? OR 93? OR 94? OR 95? OR 96? OR 97? OR 98? OR 99? OR 10?? OR 11?? OR 12?? OR 13?? OR 14?? OR 15?? OR 16?? OR 17?? OR 18?? OR 19?? OR 20?? OR 21?? OR 22?? OR 23?? OR 24?? OR 25?? OR 26?? OR 27?? OR 28?? OR 29?? OR 30?? OR 31?? OR 32?? OR 33?? OR 34?? OR 35?? OR 36?? OR 37?? OR 38?? OR 39?? OR 40?? OR 41?? OR 42?? OR 43?? OR 44?? OR 45?? OR 46?? OR 47?? OR 48?? OR 49?? OR 50?? OR 51?? OR 52?? OR 53?? OR 54?? OR 55?? OR 56?? OR 57?? OR 58?? OR 59?? OR 60?? OR 61?? OR 62?? OR 63?? OR 64?? OR 65?? OR 66?? OR 67?? OR 68?? OR 69?? OR 70?? OR 71?? OR 72?? OR 73?? OR 74?? OR 75?? OR 76?? OR 77?? OR 78?? OR 79?? OR 80?? OR 81?? OR 82?? OR 83?? OR 84?? OR 85?? OR 86?? OR 87?? OR 88?? OR 89?? OR 90?? OR 91?? OR 92?? OR 93?? OR 94?? OR 95?? OR 96?? OR 97?? OR 98?? OR 99??) NEXT/6 (patients OR patient-record* OR patient-file* OR subjects OR individuals OR cases OR persons OR men OR women OR males OR females OR participant* OR people OR children OR adolescent* OR boys OR girls OR teens OR teenagers OR infants OR newborns OR elderly OR survivor* OR specimen* OR sample* OR episode* OR isolate* OR pediatric OR paediatric OR adult* OR surgeries OR operations OR observations OR mother* OR father* OR neonate* OR volunt* OR recruit* OR infect* OR donor* OR presented OR treatments )) OR ((hundred OR thousand OR million) NEXT/5 (patients OR patient-record* OR patient-file* OR subjects OR individuals OR cases OR persons OR men OR women OR participants OR people OR children OR adolescent* OR boys OR girls OR teens OR teenagers OR infants OR newborns OR elderly OR survivor* OR specimen* OR sample* OR episode* OR isolate* OR pediatric OR paediatric OR adult* OR surgeries OR operations OR observations OR mother* OR father* OR neonate* OR volunt* OR recruit* OR infect* OR donor* OR presented OR treatments)) OR ((n OR included OR recruited OR randomized OR randomized OR assigned OR a-total-of OR group-of OR groups-of OR cohort-of OR cohorts-of) NEXT/2 (00? OR 01? OR 02? OR 03? OR 04? OR 05? OR 06? OR 07? OR 08? OR 09? OR 10? OR 11? OR 12? OR 13? OR 14? OR 15? OR 16? OR 17? OR 18? OR 19? OR 20? OR 21? OR 22? OR 23? OR 24? OR 25? OR 26? OR 27? OR 28? OR 29? OR 30? OR 31? OR 32? OR 33? OR 34? OR 35? OR 36? OR 37? OR 38? OR 39? OR 40? OR 41? OR 42? OR 43? OR 44? OR 45? OR 46? OR 47? OR 48? OR 49? OR 50? OR 51? OR 52? OR 53? OR 54? OR 55? OR 56? OR 57? OR 58? OR 59? OR 60? OR 61? OR 62? OR 63? OR 64? OR 65? OR 66? OR 67? OR 68? OR 69? OR 70? OR 71? OR 72? OR 73? OR 74? OR 75? OR 76? OR 77? OR 78? OR 79? OR 80? OR 81? OR 82? OR 83? OR 84? OR 85? OR 86? OR 87? OR 88? OR 89? OR 90? OR 91? OR 92? OR 93? OR 94? OR 95? OR 96? OR 97? OR 98? OR 99? OR 10?? OR 11?? OR 12?? OR 13?? OR 14?? OR 15?? OR 16?? OR 17?? OR 18?? OR 19?? OR 20?? OR 21?? OR 22?? OR 23?? OR 24?? OR 25?? OR 26?? OR 27?? OR 28?? OR 29?? OR 30?? OR 31?? OR 32?? OR 33?? OR 34?? OR 35?? OR 36?? OR 37?? OR 38?? OR 39?? OR 40?? OR 41?? OR 42?? OR 43?? OR 44?? OR 45?? OR 46?? OR 47?? OR 48?? OR 49?? OR 50?? OR 51?? OR 52?? OR 53?? OR 54?? OR 55?? OR 56?? OR 57?? OR 58?? OR 59?? OR 60?? OR 61?? OR 62?? OR 63?? OR 64?? OR 65?? OR 66?? OR 67?? OR 68?? OR 69?? OR 70?? OR 71?? OR 72?? OR 73?? OR 74?? OR 75?? OR 76?? OR 77?? OR 78?? OR 79?? OR 80?? OR 81?? OR 82?? OR 83?? OR 84?? OR 85?? OR 86?? OR 87?? OR 88?? OR 89?? OR 90?? OR 91?? OR 92?? OR 93?? OR 94?? OR 95?? OR 96?? OR 97?? OR 98?? OR 99??)) OR large* NEXT cohort* OR population-based OR multi-register* OR multiregister* OR regist* NEXT based OR multicent* OR multi-cent* OR national-stud* OR national-regist*):ab,ti

# **Supplemental Figure 1**. Search Results and PRISMA flowchart


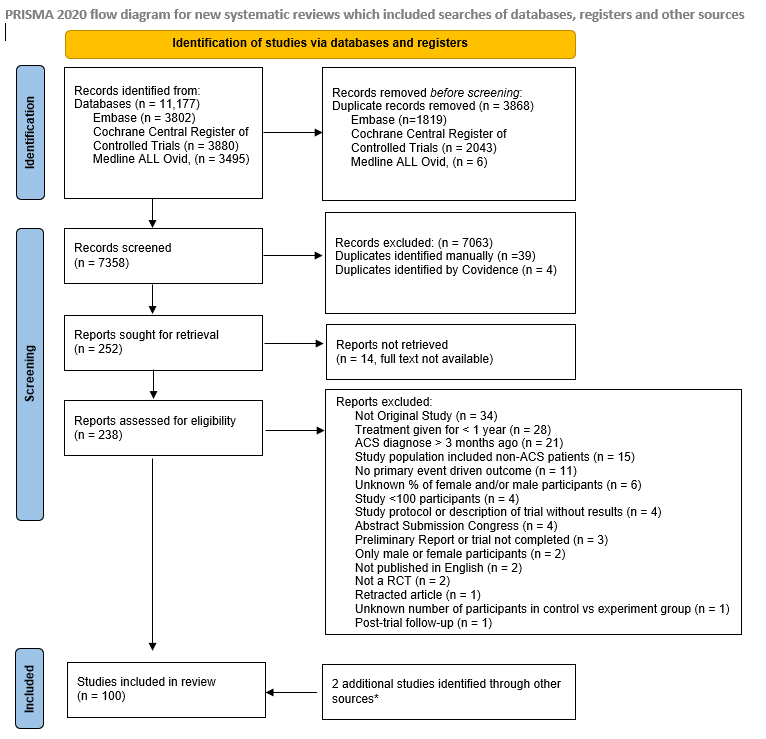


# **Supplemental Figure 2.** Risk of bias summary

# **Supplemental Figure 3**. Funnel plot of the meta-analysis of sex differences


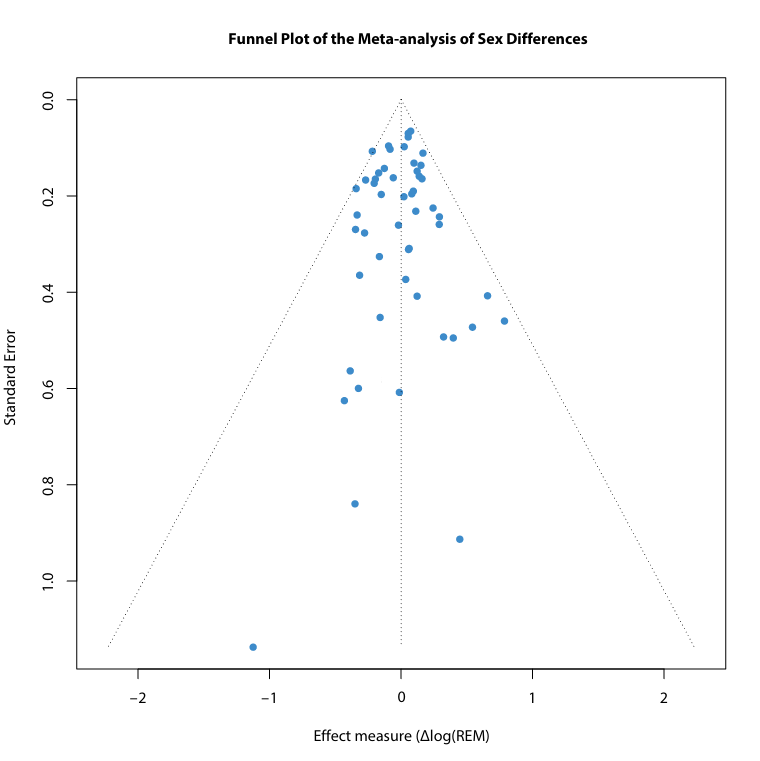


| **Author** | **Year** | **Sex-specific criteria:** | **Trial Medication** | | **Endpoint** | **Follow-up** | **Age *^)^ = range** | | **Females** | **Overall treatment effect (REM)** | | | |
| --- | --- | --- | --- | --- | --- | --- | --- | --- | --- | --- | --- | --- | --- |
|  |  |  | **Exp** | **Ctrl** |  | **^*)^  = terminated early** | **Exp** | **Ctrl** | **%** | **Measure**  **^*)^ = calc.** | **Estimate** | **LL** | **UU** |
| **Anti-thrombotic treatment** | | | | | | | | | | | | | |
| Breddin et al. | 1980 | NO | Acetylsalicyclic Acid | Placebo | ACM | Scheduled 2 years | x | x | 22 | OR* | 0.81 | 0.47 | 1.38 |
|  |  |  | Phenprocoumon | Placebo |  |  | x | x | 22 | OR* | 1.20 | 0.73 | 1.97 |
| Cairns et al. | 1985 | NO | Aspirin | Placebo | Cardiac mortality or nonfatal MI | 18 months | 56.7 | 57 | 27 | OR* | 0.77 | 0.38 | 1.56 |
|  |  |  | Sulfinpyrazone | Placebo |  |  | 57.8 | x | 27 | OR* | 1.05 | 0.54 | 2.04 |
|  |  |  | Aspirin + Sulfinpyrazone | Placebo |  |  | 56.6 | x | 27 | OR* | 0.62 | 0.30 | 1.31 |
| ASPECT Research Group | 1994 | Pregnancy | Nicoumalone or phenprocoumon | Placebo | ACM | 37 months | 61 (11) | 61 (11) | 20.0 | HR | 0.90 | 0.73 | 1.11 |
| Condorelli et al. | 1994 | NO | Sulodexide | SOC | ACM | Scheduled for minimum 12 months | 59.3 ± 9.8 | 60.1±9.9 | 18 | OR* | 0.66 | 0.51 | 0.86 |
| CARS Investigators | 1997 | Women of childbearing potential | Low dose warfarin + low dose aspirin | High dose aspirin | CV mortality, reinfarction or non-fatal ischaemic stroke | 14 months | x | x | 23 | RR* | 1.03 | 0.85 | 1.22 |
|  |  |  | High dose warfarin + low dose aspirin | High dose aspirin |  |  | x | x | 23 | RR | 0.95 | 0.81 | 1.12 |
| Ishikawa et al. | 1997 | NO | Antiplatelet agent | No antiplatelet agent | Congestive HF or sudden mortality, recurrent MI | 12.5 months | 60.1±11.2 | 60.0±11.8 | 22 | OR | 0.40 | 0.23 | 0.71 |
| Yasue et al. | 1999 | NO | Aspirin | No antiplatelets | CV mortality, reinfarction, uncontrolled UA requiring hospitalization, and nonfatal ischemic stroke | 475 days | 65.0 ± 0.7 | 65.5 ± 0.8 | 30 | OR | 0.79 | 0.53 | 1.19 |
|  |  |  | Trapidil | No antiplatelets |  |  | 65.2 ± 0.7 | x | 30 | OR | 0.50 | 0.31 | 0.80 |
| Huynh et al. | 2001 | Women of childbearing potential | Aspirin + warfarin | Warfarin + Placebo | ACM, MI, UA requiring hospitalization | Scheduled 13 months | 66±12 | 67±12 | 20 | OR* | 0.78 | 0.22 | 2.74 |
|  |  |  | Aspirin + warfarin | Aspirin + Placebo |  |  | 66±12 | 68±11 | 20 | OR* | 0.99 | 0.27 | 3.63 |
| Hurlen et al. | 2002 | NO | Warfarin | Aspirin | ACM, nonfatal reinfarction, or thromboembolic cerebral stroke | 4 years | 59.7±9.9 | 60.7±9.7 | 24 | HR | 0.81 | 0.69 | 0.95 |
| Herlitz et al. | 2004 | NO | Warfarin + aspirin | Aspirin | CV mortality, reinfarction or stroke | 5.0 years | 66±11 | 66±11 | 27 | OR* | 0.96 | 0.89 | 1.12 |
| Wallentin et al. | 2009 | Pregnancy or lactation | Ticagrelor | Clopidogrel | CV mortality, MI, or stroke | Scheduled 12 months | 62.0 | 62.0 | 28 | HR | 0.84 | 0.77 | 0.92 |
| Alexander et al. | 2011 | Pregnancy, lactation, or unwillingness to use medically accepted contraception | Apixaban | Placebo | CV mortality, MI, or ischemic stroke | 241 days* | 67 (59-73) | 67 (58-74) | 32 | HR | 0.95 | 0.8 | 1.11 |
| Tricoci et al. | 2012 | Pregnancy, lactation, or unwillingness to use medically accepted contraception | Vorapaxar | Placebo | CV mortality, MI, stroke, recurrent ischemia with rehospitalization, or urgent coronary revascularization | 502 days* | 64.0 (58-71) | 64.0 (58-72) | 28 | HR | 0.92 | 0.85 | 1.01 |
| Roe et al. | 2012 | NO | Prasugrel | Clopidogrel | CV mortality, nonfatal MI or stroke | 17 months | 62 (56–68) | 62 (56–68) | 36 | HR | 0.91 | 0.79 | 1.05 |
| Mega et al. | 2012 | NO | Rivaroxaban 2.5 mg | Placebo | CV mortality, MI or stroke | Maximum 31 months | 61.8±9.2 | 61.5±9.4 | 25 | HR | 0.84 | 0.74 | 0.96 |
|  |  |  | Rivaroxaban 5 mg |  |  |  | 61.9±9.0 | x | X | x | x | x | x |
| Zhu et al. | 2015 | Pregnancy | Individualized antiplatelet therapy: low responsiveness 6M cilostazol | SOC | CV mortality, MI or stroke | Scheduled 12 months | 60.2 ± 10.9 | 60.1 ± 10.9 | 34 | OR* | 0.61 | 0.25 | 1.45 |
| Wang et al. | 2016 | NO | Ticagrelor | Clopidogrel | CV mortality, MI or stroke | Scheduled 12 months | 79 (76–85) | 80 (74–86) | 33 | HR | 0.47 | 0.23 | 0.98 |
| Sibbing et al. | 2017 | Pregnancy, lactation, or less than 90 days postpartum or unwillingness to use medically accepted contraception | Guided de-escalation | Prasugrel 12 months | CV mortality, MI, stroke or bleeding | Scheduled 12 months | 59.0±10.1 | 58.5±10.2 | 21 | HR | 0.81 | 0.62 | 1.06 |
| Cuisset et al. | 2017 | Pregnancy | Switched DAPT | Unchanged DAPT | CV mortality, urgent revascularization, stroke and bleeding | 355 days | 60.6 ± 10.2 | 59.6 ± 10.3 | 18 | HR | 0.48 | 0.34 | 0.68 |
| Hahn et al. | 2018 | NO | 6M-DAPT | 12M- or longer DAPT | ACM, MI, or stroke | Scheduled 18 months | 62.0±11.5 | 62.2±11.9 | 25 | HR | 1.13 | 0.79 | 1.62 |
| Notarangelo et al. | 2018 | NO | Genotype-guided treatment | SOC | CV mortality, nonfatal MI or stroke, and major bleeding | Scheduled 12 months* | 71.1 ± 12.3 | 70.7 ± 12.1 | 32 | HR | 0.58 | 0.43 | 0.78 |
| Savonitto et al. | 2018 | NO | Prasugrel | Clopidogrel | ACM, MI, disabling stroke, and rehospitalization for CV causes or bleeding | 12 months* | 80 (77–84) | 80 (77–84) | 40 | HR | 1.01 | 0.78 | 1.30 |
| Wu et al. | 2018 | NO | Ticagrelor | Clopidogrel | CV mortality, recurrent angina or MI, stroke HF, in-stent thrombosis or thromboembolic events | Scheduled 12 months | 59.0 ± 10.2 | 61.1 ± 11.6 | 21 | OR* | 0.23 | 0.11 | 0.52 |
| Claassens et al. | 2019 | Pregnancy, lactation, or less than 90 days postpartum | Genotype-guided (ticagrelor/prasugrel or clopidogrel) | SOC | ACM, MI, stent thrombosis, stroke, or major bleeding | Minimum 12 months | 61.9±11.1 | 61.4±11.5 | 25 | HR | 0.87 | 0.62 | 1.21 |
| DeLuca et al. | 2019 | Pregnancy | 3M-DAPT | 12M-DAPT | ACM, MI, stent thrombosis, stroke, target vessel revascularisation and bleeding | Scheduled 2 years | 61.0 (53.0-69.0) | 60.0 (52.0-68.0) | 20 | HR | 0.96 | 0.71 | 1.29 |
| Schupke et al. | 2019 | Pregnancy, lactation, or less than 90 days postpartum or unwillingness to use medically accepted contraception | Ticagrelor | Prasugrel | ACM, MI, or stroke | Scheduled 12 months | 64.5±12.0 | 64.6±12.1 | 24 | HR | 1.36 | 1.09 | 1.7 |
| Gimbel et al. | 2020 | NO | Clopidogrel | Ticagrelor / prasugrel | ACM, MI, stroke, PLATO major and minor bleeding | Scheduled 12 months | 77 (73–81) | 77 (73–82) | 36 | HR | 0.82 | 0.66 | 1.03 |
| Kim et al. | 2020 | Women of childbearing potential/negative pregnancy test | De-escalation | Conventional | ACM, non-fatal MI, stent thrombosis, repeat revascularisation, stroke, and bleeding | Scheduled 12 months | 58.7±9.0 | 58.9±9.1 | 11 | HR | 0.70 | 0.52 | 0.92 |
| You et al. | 2020 | NO | HPR switch: ticagrelor | HPR nonswitch: clopidogrel | ACM, nonfatal MI, target vessel revascularization, and ischemic stroke | Scheduled 12 months | 66.29 ± 8.55 | 68.62 ± 9.41 | 35 | OR* | 0.39 | 0.18 | 0.84 |
| Zhang et al. | 2020 | NO | Individualized antiplatelet therapy | Clopidogrel | ACM, non-fatal MI and definite stent thrombosis | Scheduled 12 months | 63.6 ± 10.7 | 64.6 ± 10.7 | 30 | OR* | 0.37 | 0.17 | 0.81 |
| Al-Rubaish et al. | 2021 | Pregnancy and lactation | Genotype-guided (clopidogrel or ticagrelor) | SOC | CV mortality, MI, non-fatal stroke or major bleeding | Scheduled minimum 12 months | 56.74 ± 11.84 | 55.47 ± 11.22 | 19 | HR | 7.14 | 4.96 | 10.3 |
| Kim et al. | 2021 | Pregnancy, lactation, or less than 90 days postpartum or unwillingness to use medically accepted contraception | Clopidogrel + aspirin | Ticagrelor + aspirin | CV mortality, MI, stroke, and bleeding. | Scheduled 12 months | 60.1±11.3 | 59.9±11.4 | 17 | HR | 0.55 | 0.4 | 0.76 |
| Shi et al. | 2021 | Pregnancy or lactation | Genotype-guided treatment | SOC | ACM, MI, stroke, urgent coronary revascularization, and stent thrombosis | Scheduled 12 months | 59.7±9.6 | 59.8±10.4 | 25 | HR | 0.28 | 0.10 | 0.77 |
| Watanabe et al. | 2022 | NO | ½M-DAPT | 12M-DAPT | CV mortality, MI, any stroke or definite stent thrombosis | Scheduled 12 months | 67.0±11.9 | 66.6±11.9 | 21 | HR | 1.14 | 0.8 | 1.62 |
| Park et al. | 2023 | Pregnancy | 1M-TAPT | 12M-DAPT | ACM, recurrent MI, stroke or repeat revascularization | Scheduled 12 months | 61.4 ± 12.9 | 62.6 ± 12.0 | 18 | HR | 1.30 | 0.79 | 2.14 |
|  |  |  | 6M-TAPT | 12M-DAPT |  |  | 60.9 ± 12.5 | x | 18 | HR | 1.36 | 0.83 | 2.23 |
| Hong et al. | 2024 | Women of childbearing potential | 1M-DAPT + ticagrelor monotherapy | 12M-DAPT (ticagrelor) | ACM, MI, stent thrombosis, stroke, and major bleeding | Scheduled 12 months | 61±10 | 61±10 | 17 | HR | 0.54 | 0.37 | 0.8 |
| Gao et al. | 2025 | Pregnant or lactating | Stepwise DAPT de-escalation | Standard DAPT | ACM, stroke, MI, revascularisation and bleeding | Scheduled 12 months | 59.4 ± 10.7 | 59.0 ± 11.0 | 25 | HR | 1.04 | 0.77 | 1.40 |
| Jang et al. | 2025 | Women of childbearing potential who plan to become pregnant within the study period | 1M-DAPT | 12M-DAPT | ACM, non-fatal MI, stroke, ischaemia-driven target vessel revascularisation, and bleeding | Scheduled 12 months | 60.4±9.8 | 61.3±9.6 | 17 | HR | 0.51 | 0.27 | 0.95 |
| **Lipid-lowering treatment** | | | | | | | | | | | | | |
| Nilsen et al. | 2001 | NO | N3 fatty acids | Corn oil | CV mortality, resuscitation, recurrent MI, or UA | Median 1.5 years | 64.4 | 63.6 | 21 | HR | 1.19 | 0.76 | 1.86 |
| Liem et al. | 2002 | NO | Fluvastatin | Placebo | ACM, recurrent AMI, recurrent ischaemia necessitating hospitalization or revascularization or ischemia on ECG at 12 months | Scheduled 12 months | 61 (30–86) | 60 (32–87) | 17 | OR* | 0.86 | 0.59 | 1.25 |
| Cannon et al. | 2004 | NO | Atorvastatin | Pravastatin | ACM, MI, UA requiring hospitalization, revascularization, and stroke | 24 months | 58.1±11.2 | 58.3±11.3 | 22 | HR* | 0.85 | 0.73 | 0.95 |
| Sakamoto et al. | 2006 | NO | Statin | No statin | CV mortality, nonfatal AMI, recurrent symptomatic myocardial ischemia, HFH, and nonfatal stroke | 416 days | 63 ± 11 | 65 ± 12 | 28 | HR | 1.94 | 1.07 | 3.67 |
| Chi H, Wang S, Chen J, Zhang J. | 2007 | NO | Simvastatin | SOC, no statins | Cardiac mortality, HF, rehospitalization for aggravation of CHD, and stroke | Scheduled 18 months | 63.2±8.7 | 65.4±7.8 | 42 | OR* | 0.35 | 0.14 | 0.87 |
| Rauch et al. | 2010 | Pregnancy, lactation, or unwillingness to use medically accepted contraception | Omega-3-acid ethyl esters | Placebo | Sudden CV mortality | 365 days | 64.0 (54.0–72.0) | 64.0 (54.0–72.0) | 26 | OR | 0.95 | 0.56 | 1.60 |
| Colivicchi et al. | 2010 | NO | Atorvastatin | SOC | CV mortality, non-fatal MI and disabling stroke | 258 days* | 75.2±9.9 | 73.9±9.4 | 49 | HR | 0.56 | 0.33 | 0.93 |
| Schwartz et al. | 2012 | Pregnancy, lactation, or unwillingness to use medically accepted contraception | Dalcetrapib | Placebo | Mortality from CHD, nonfatal MI, ischemic stroke, UA, or cardiac arrest with resuscitation | 31 months | 60.3±9.1 | 60.1±9.1 | 19 | HR | 1.04 | 0.93 | 1.16 |
| Izawa et al. | 2014 | Pregnancy | Pravastatin | Atorvastatin | ACM, non-fatal MI, non-fatal stroke, HFH or any type of coronary revascularization. | Minimum 24 months | 65.7±11.7 | 66.3±11.4 | 19 | HR | 1.18 | 0.86 | 1.62 |
| O’Donoghue et al. | 2014 | Pregnancy or lactation | Darapladib | Placebo | CHD mortality, MI, or revascularization for myocardial ischemia | 2.5 years | 64 (59-70) | 64 (59-71) | 26 | HR | 1.00 | 0.91 | 1.09 |
| Cannon et al. | 2015 | Pregnancy, lactation, or unwillingness to use medically accepted contraception | Simvastatin + ezetimibe | Simvastatin and placebo | CV mortality, nonfatal MI, UA requiring rehospitalization, coronary revascularization (≥30 days after randomization), or nonfatal stroke | 6 years | 63.6±9.7 | 63.6±9.8 | 24 | HR | 0.94 | 0.89 | 0.99 |
| Liu et al. | 2016 | NO | Atorvastatin (high intensity) | Atorvastatin (moderate intensity) | CV mortality, spontaneous MI, unplanned revascularization | Scheduled 12 months | 61.6 ± 8.7 | 62.1 ± 10.2 | 51 | HR | 0.61 | 0.36 | 0.91 |
| Nosaka et al. | 2017 | NO | Pitavastatin + EPA | Pitavastatin | CV mortality, nonfatal stroke, nonfatal MI and revascularization | Scheduled 12 months | 70 ± 11 | 71 ± 12 | 24 | HR | 0.42 | 0.21 | 0.87 |
| Hagiwara et al. | 2017 | Pregnancy or lactation | Pitavastatin + ezetimibe (target LDL-C < 70 mg/dL) | Pitavastatin (target LDL-C 90 mg/dL to 100 mg/dL) | ACM, non-fatal MI, non-fatal stroke, UA, or revascularization | 3.9 years | 65.7 ± 11.7 | 65.5 ± 11.9 | 24 | HR | 0.89 | 0.76 | 1.04 |
| Liu et al. | 2017 | NO | Atorvastatin + ezetimibe | Atorvastain (double-dose) | Cardiac mortality, spontaneous MI, unplanned revascularization | Scheduled 12 months | 84.2±2.9 | 84.0±1.8 | 48 | HR | 1.12 | 0.51 | 2.55 |
| Tardif et al. | 2022 | Pregnancy, lactation, or unwillingness to use medically accepted contraception | Dalcetrapib | Placebo | CV mortality, resuscitated cardiac arrest, non-fatal MI, or non-fatal stroke | 39.9 months | 62.2±9.2 | 62.3 ±9.3 | 23 | HR | 0.88 | 0.75 | 1.03 |
| **Beta-blockers** | | | | | | | | | | | | | |
| Green KG et al. | 1975 | NO | Practolol | Placebo | ACM, non-fatal reinfarction | 14.2 months* | 55 | 55 | 14 | OR* | 0.69 | 0.54 | 0.88 |
| Ahlmark G, Saetre H | 1976 | NO | Alprenolol | SOC | ACM | 2 years | 57.7 | 56.4 | 12 | OR* | 0.46 | 0.16 | 1.34 |
| Barber et al. | 1976 | NO | Practolol | Placebo | ACM | Scheduled 2 years | 62 | 63 | 39 | OR* | 0.82 | 0.50 | 1.35 |
| The norwegian multicenter Study Group | 1981 | NO | Timolol | Placebo | ACM | 17 months | x | x | 21 | OR* | 0.6 | 0.46 | 0.79 |
| Julian et al. | 1982 | Pregnancy | Sotalol | Placebo | ACM | Scheduled 12 months | 55.2± 7.9 | 55.4± 7.9 | 21 | OR* | 0.81 | 0.55 | 1.18 |
| The European Infarction Study Group | 1984 | NO | Oxprenolol | Placebo | ACM | Scheduled 12 months | 54.7 | 54.9 | 16 | OR* | 1.33 | 0.89 | 1.98 |
| Lopressor Intervention Trial Research Group | 1987 | NO | Metoprolol | Placebo | ACM | Scheduled minimum 12 months* | 58 | 58 | 17 | OR* | 1.06 | 0.74 | 1.51 |
| Navarro-Lopez et al. | 1993 | NO | Amiodarone | No antiarrhythmic treatment | ACM or cardiac arrest with resuscitation | Median 2.8 years | 58 ± 10 | 57 ± 9 | 10 | OR* | 0.46 | 0.14 | 1.53 |
|  |  |  | Metoprolol | No antiarrhythmic treatment |  |  | 59 ± 10 | x | 10 | OR* | 1.91 | 0.82 | 4.45 |
| CAPRICORN Investigators | 2001 | Pregnancy, lactation or planned pregnancy | Carvedilol | Placebo | ACM or CV readmission | 1.3 years | 63 (39-88)^#^ | 63 (25-90)^#^ | 26 | HR | 0.92 | 0.8 | 1.07 |
| Torp-Pedersen et al. | 2002 | Pregnancy, lactation, or unwillingness to use medically accepted contraception | Bucindolol | Placebo | ACM | 225 days* | 67 (32–92) | 70 (29–92) | 27 | HR | 0.88 | 0.5 | 1.5 |
| The Japanese β-blockers and Calcium Antagonists Myocardial Infarction (JBCMI) Investigators | 2004 | NO | Beta-blockers | Calcium antagonists | CV mortality, nonfatal reinfarction, uncontrolled UA, and nonfatal stroke. | 455 days | 64.0 ± 11.6 | 64.8 ± 11.3 | 21 | OR* | 1.1 | 0.78 | 1.55 |
| Mrdovic et al. | 2007 | NO | Carvedilol | Metoprolol | ACM, rehospitalizaiton for CV event,PCI or CABG, postinfarction AP, HFH | 13.4 months | 60.5 ± 10.4 | 62.9 ± 10.5 | 32 | OR* | 0.29 | 0.17 | 0.51 |
| Ozaydin et al. | 2016 | NO | Metoprolol | Nebivolol | CV mortality, nonfatal MI, HFH, hospitalization for UA, stroke or revascularization | Scheduled 12 months | 59±13 | 61±11 | 16 | OR* | 2.5 | 0.98 | 6.39 |
|  |  |  | Carvedilol | Nebivolol |  |  | 59±12 | x | 16 | OR* | 1.47 | 0.55 | 3.92 |
| Watanabe et al. | 2018 | NO | Carvedilol | No beta-blocker | ACM, MI, HFH, hospitalization for ACS | 3.9 years | 63.9±11.2 | 64.5±11.3 | 20 | HR | 0.75 | 0.47 | 1.16 |
| Yndigegn et al. | 2024 | NO | Beta-blockers | No beta-blockers | ACM, MI | 3.5 years | 65 (57–73) | 65 (57–73) | 23 | HR | 0.96 | 0.79 | 1.16 |
| **RAAS-inhibition** | | | | | | | | | | | | | |
| Pfeffer et al. | 1992 | Women of childbearing potential who are unwilling to use medically accepted contraception | Captopril | Placebo | ACM | 42 months | 59.3 | 59.5 | 18 | OR* | 0.79 | 0.64 | 0.96 |
| AIRE Study Investigators | 1993 | NO | Ramipril | Placebo | ACM | 15 months | 64.9 (10.8) | 65.1 (10.8) | 26 | HR | 0.73 | 0.6 | 0.89 |
| Kober et al. | 1995 | Pregnancy or lactation | Trandolapril | Placebo | ACM | 24-50 months | 67.7 | 67.3 | 29 | RR | 0.78 | 0.67 | 0.91 |
| Pfeffer et al. | 2003 | Pregnancy, lactation, or less than 90 days postpartum or unwillingness to use medically accepted contraception | Valsartan | Captopril | ACM | 24.7 months | 65.0±11.8 | 64.9±11.8 | 31 | HR | 1.00 | 0.92 | 1.09 |
|  |  |  | Valsartan + captopril | Captopril |  |  | 64.6±11.9 | 64.9±11.8 | 31 | HR | 0.98 | 0.913 | 1.05 |
| Pitt et al. | 2003 | NO | Eplerenone | Placebo | ACM | 16 months | 64±11 | 64±12 | 29 | RR | 0.85 | 0.75 | 0.96 |
| Ueshima et al. | 2004 | Pregnancy or possible pregnancy | Enalapril / captopril / cilazapril | Non-ACEI | ACM, non-fatal MI, coronary revascularization, HFH or angina recuiring hospitalization | 5.8 years | 62.2 ± 10.4 | 62.4 ± 11.0 | 22 | OR* | 1.00 | 0.75 | 1.33 |
| Pfeffer et al. | 2021 | Pregnancy, lactation, or unwillingness to use medically accepted contraception | Sacubitril–Valsartan | Ramipril | CV mortality or incidence HF | 22 months | 64.0±11.6 | 63.5±11.4 | 24 | HR | 0.90 | 0.78 | 1.04 |
| **Others** | | | | | | | | | | | | | |
| Lovell RRH et al. | 1971 | NO | Phenytoin high dose | Phenytoin low dose | ACM | Scheduled 12 months | x | x | 12 | OR* | 0.88 | 0.49 | 1.58 |
| Peter et al. | 1978 | NO | Phenytoin | SOC | ACM | Scheduled 24 months | x | x | 19 | OR* | 1.42 | 0.65 | 3.13 |
| The Anturane Reinfarction Trial Research Group | 1978 | NO | Sulfinpyrazone | Placebo | CV mortality | 8.4 months | 56.8 | 56.6 | 14 | OR* | 0.54 | 0.32 | 0.89 |
| Moss et al. | 1988 | Women of childbearing potential who are unwilling to use medically accepted contraception | Diltiazem | Placebo | CV mortality, nonfatal reinfarction | 25 months | 58±10 | 58±10 | 20 | HR | 0.90 | 0.74 | 1.08 |
| The Danish Study Group on Verapamil in Myocardial Infarction | 1990 | NO | Verapamil | Placebo | ACM | 16 months | x | x | 20 | HR | 0.8 | 0.61 | 1.05 |
| Burkart et al. | 1990 | NO | Quinidine or mexiletine | Without antiarrhythmic therapy | ACM | Scheduled 12 months | 60±8 | 61±6 | 14 | OR* | 0.73 | 0.31 | 1.72 |
|  |  |  | Amiodarone | Without antiarrhythmic therapy | |  | 61±7 | 61±6 | 14 | OR* | 0.35 | 0.12 | 1.02 |
| Ceremuzynski et al. | 1992 | NO | Amiodarone | Placebo | ACM | Scheduled 12 months | 59.4 ± 12.3 | 58.6 ± 11.8 | 30 | OR | 0.62 | 0.35 | 1.08 |
| Galloe et al. | 1993 | NO | Magnesium | Placebo | Sudden death, reinfarction, CABG | Scheduled 12 months | 67.3 | 67.8 | 38 | RR | 1.40 | 0.98 | 2.00 |
| Cairns et al. | 1997 | Women of childbearing potential | Amiodarone | Placebo | Mortality from arrhythmia or resuscitated VF | 1.8 years | 64 (31-91)^#^ | 64 (28-88)^#^ | 18 | RR* | 0.62 | 0.37 | 1.02 |
| Julian et al. | 1997 | Women of childbearing potential who are unwilling to use medically accepted contraception | Amiodarone | Placebo | ACM | 21 months | 59.6 (9.7) | 60.2 (9.2) | 16 | RR | 0.99 | 0.76 | 1.31 |
| Kober et al. | 2000 | Women of childbearing potential | Dofetilide | Placebo | ACM | Minimum 12 months | 68 (34–89)^#^ | 69 (33–92)^#^ | 26 | OR* | 0.94 | 0.76 | 1.17 |
| Liem et al. | 2004 | NO | Folic acid | no folic acid | ACM, recurrent MI, strokes, and unplanned invasive coronary interventions. | Scheduled 12 months | 59 | 59 | 30 | OR* | 0.97 | 0.58 | 1.60 |
| Cannon et al. | 2005 | NO | Gatifloxacin | Placebo | ACM, MI, UA requiring hospitalization, PCI or CABG, or stroke; | 2 years | 58.1±11.2 | 58.3±11.2 | 22 | HR | 0.95 | 0.84 | 1.08 |
| Bonaa et al. | 2006 | NO | Folic acid + Vitamin B12 + Vitamin B6 | Placebo | Mortality from CAD, recurrent MI, or stroke | 40 months | 63.6±11.9 | 62.6±11.4 | 52 | HR | 1.22 | 1.00 | 1.5 |
| Morrow et al. | 2007 | Pregnancy, lactation, or unwillingness to use medically accepted contraception. | Ranolazine | Placebo | CV mortality, MI or recurrent ischemia | 348 days | 64 (55-72) | 64 (56-72) | 35 | HR | 0.92 | 0.83 | 1.02 |
| Armitage et al. | 2010 | Pregnancy, lactation, or unwillingness to use medically accepted contraception. | Folic acid + Vitamin B12 | Placebo | Mortality from CHD, nonfatal MI, stroke, or any arterial revascularization | 2.5 years | x | x | 17 | RR | 1.04 | 0.97 | 1.12 |
| Hanash et al. | 2012 | Pregnancy, lactation or unwillingness to use medically accepted contraception | Escitalopram | Placebo | ACM, recurrent ACS, and unplanned revascularization | Scheduled 12 months | 65.3 ± 12.1 | 64.2 ± 12.2 | 37 | RR | 0.90 | 0.6 | 1.28 |
| White et al. | 2013 | Pregnancy, lactation, or unwillingness to use medically accepted contraception. | Alogliptin | Placebo | CV mortality, nonfatal MI or stroke | 18 months | 61.0 | 61.0 | 32 | HR | 0.96 | 0.82 | 1.13 |
| Lincoff et al. | 2014 | Pregnancy, lactation, or unwillingness to use medically accepted contraception | Aleglitazar | Placebo | CV mortality, nonfatal MI or stroke | 104 weeks* | 61±10 | 61±10 | 27 | HR | 0.96 | 0.83 | 1.11 |
| Yun et al. | 2016 | Women on HRT | Acarbose | No acarbose | CV mortality, nonfatal reinfarction, new-onset angina, cerebral stroke, and severe HF | 2.3 years | 62.24 ± 5.16 | 61.62 ± 4.58 | 40 | OR* | 0.4 | 0.19 | 0.83 |
| Tardif et al. | 2019 | Pregnancy, lactation, or unwillingness to use medically accepted contraception. | Colchicine | Placebo | CV mortality, resuscitated cardiac arrest, MI, stroke, or urgent coronary revascularization | 22.6 months | 60.6±10.7 | 60.5±10.6 | 19 | HR | 0.77 | 0.61 | 0.96 |
| Tong et al | 2020 | Pregnancy, lactation, or unwillingness to use medically accepted contraception. | Colchicine | Placebo | ACM, ACS, urgent revascularization, and noncardioembolic ischemic stroke | 371 days | 59.7±10.2 | 60.0±10.4 | 21 | HR | 0.65 | 0.38 | 1.09 |
| Ray et al. | 2020 | Pregnancy or unwillingness to use medically accepted contraception. | Apabetalone | Placebo | CV mortality, nonfatal MI, or stroke | 26.5 months | 62 (55-68) | 62 (56-68) | 25 | HR | 0.82 | 0.65 | 1.04 |
| Brie et al. | 2022 | Pregnancy. | Pentoxifylline | Placebo | ACM, ACS, ischemic-driven revascularization or stroke | 20 months | 62.3±10.7 | 61.8±10.2 | 20 | RR | 0.78 | 0.49 | 1.26 |
| Butler et al. | 2024 | Pregnancy, lactation, or unwillingness to use medically accepted contraception. | Empagliflozin | Placebo | ACM, HFH | 17.9 months | 63.6 ± 11.0 | 63.7 ± 10.8 | 25 | HR | 0.9 | 0.76 | 1.06 |
| Li et al. | 2025 | NO | Ivabradine | SOC | CV mortality | Scheduled 12 months | 68.0 ± 10.3 | 66.3 ± 10.3 | 39 | HR | 1.03 | 0.79 | 1.26 |

## **Supplemental Table 1.** Trial and patient characteristics of all included trials (1-102)

**Abbreviations used:** Exp = experimental; Ctrl = control; REM = relative effect measure; LL = lower limit; UL = upper limit; ACM = all-cause mortality; OR = odds ratio; MI = myocardial infarction; DAPT = dual anti-platelet therapy; HR = hazards ratio; SOC = standard of care; CV = cardiovascular; RR = relative risk; HF = heart failure; UA = unstable angina; HPR = high platelet reactivity; TAPT = triple anti-platelet therapy; HFH = heart failure hospitalization

**References:**
1. Cannon CP, Blazing MA, Giugliano RP, McCagg A, White JA, Theroux P, et al. Ezetimibe Added to Statin Therapy after Acute Coronary Syndromes. N Engl J Med. 2015;372(25):2387-97.

2. Roe MT, Armstrong PW, Fox KA, White HD, Prabhakaran D, Goodman SG, et al. Prasugrel versus clopidogrel for acute coronary syndromes without revascularization. N Engl J Med. 2012;367(14):1297-309.

3. Tricoci P, Huang Z, Held C, Moliterno DJ, Armstrong PW, Van de Werf F, et al. Thrombin-receptor antagonist vorapaxar in acute coronary syndromes. N Engl J Med. 2012;366(1):20-33.

4. Alexander JH, Lopes RD, James S, Kilaru R, He Y, Mohan P, et al. Apixaban with antiplatelet therapy after acute coronary syndrome. N Engl J Med. 2011;365(8):699-708.

5. Anonymous. The Lopressor Intervention Trial: multicentre study of metoprolol in survivors of acute myocardial infarction. Lopressor Intervention Trial Research Group. Eur Heart J. 1987;8(10):1056-64.

6. Ahlmark G, Saetre H. Long-term treatment with beta-blockers after myocardial infarction. Eur J Clin Pharmacol. 1976;10(2):77-83.

7. Izawa A, Kashima Y, Miura T, Ebisawa S, Kitabayashi H, Yamamoto H, et al. Assessment of lipophilic vs. hydrophilic statin therapy in acute myocardial infarction: ALPS-AMI study. Circ J. 2014;79(1):161-8.

8. Shi X, Zhang Y, Zhang Y, Zhang R, Lin B, Han J, et al. Personalized Antiplatelet Therapy Based on CYP2C19 Genotypes in Chinese ACS Patients Undergoing PCI: A Randomized Controlled Trial. Front Cardiovasc Med. 2021;8.

9. Yun P, Du AM, Chen XJ, Liu JC, Xiao H. Effect of Acarbose on Long-Term Prognosis in Acute Coronary Syndromes Patients with Newly Diagnosed Impaired Glucose Tolerance. J Dia Res. 2016;2016.

10. Wang H, Wang X. Efficacy and safety outcomes of ticagrelor compared with clopidogrel in elderly Chinese patients with acute coronary syndrome. Ther Clin Risk Manage. 2016;12:1101-5.

11. Ozaydin M, Yucel H, Kocyigit S, Adali MK, Aksoy F, Kahraman F, et al. Nebivolol versus Carvedilol or Metoprolol in Patients Presenting with Acute Myocardial Infarction Complicated by Left Ventricular Dysfunction. Med Princ Pract. 2016;25(4):316-22.

12. Cairns JA, Connolly SJ, Roberts R, Gent M. Randomised trial of outcome after myocardial infarction in patients with frequent or repetitive ventricular premature depolarisations: CAMIAT. Canadian Amiodarone Myocardial Infarction Arrhythmia Trial Investigators. Lancet (london, england). 1997;349(9053):675-82.

13. Al-Rubaish AM, Al-Muhanna FA, Alshehri AM, Al-Mansori MA, Alali RA, Khalil RM, et al. Bedside testing of CYP2C19 vs. conventional clopidogrel treatment to guide antiplatelet therapy in ST-segment elevation myocardial infarction patients. International journal of cardiology. 2021;343:15-20.

14. Hong SJ, Lee SJ, Suh Y, Yun KH, Kang TS, Shin S, et al. Stopping Aspirin Within 1 Month After Stenting for Ticagrelor Monotherapy in Acute Coronary Syndrome: The T-PASS Randomized Noninferiority Trial. Circulation. 2024;149(8):562-73.

15. Watanabe H, Morimoto T, Natsuaki M, Yamamoto K, Obayashi Y, Ogita M, et al. Comparison of Clopidogrel Monotherapy After 1 to 2 Months of Dual Antiplatelet Therapy With 12 Months of Dual Antiplatelet Therapy in Patients With Acute Coronary Syndrome: The STOPDAPT-2 ACS Randomized Clinical Trial. JAMA Cardiol. 2022;7(4):407-17.

16. Kim CJ, Park MW, Kim MC, Choo EH, Hwang BH, Lee KY, et al. Unguided de-escalation from ticagrelor to clopidogrel in stabilised patients with acute myocardial infarction undergoing percutaneous coronary intervention (TALOS-AMI): an investigator-initiated, open-label, multicentre, non-inferiority, randomised trial. Lancet. 2021;398(10308):1305-16.

17. Zhang M, Wang JR, Zhang Y, Zhang P, Ren MY, Jia XM, et al. Effects of individualized antiplatelet therapy based on CYP2C19 genotype and platelet function on the prognosis of patients after PCI. Eur Rev Med Pharmacol Sci. 2020;24(20):10753-68.

18. Kim HS, Kang J, Hwang D, Han JK, Yang HM, Kang HJ, et al. Prasugrel-based de-escalation of dual antiplatelet therapy after percutaneous coronary intervention in patients with acute coronary syndrome (HOST-REDUCE-POLYTECH-ACS): an open-label, multicentre, non-inferiority randomised trial. Lancet. 2020;396(10257):1079-89.

19. Gimbel M, Qaderdan K, Willemsen L, Hermanides R, Bergmeijer T, de Vrey E, et al. Clopidogrel versus ticagrelor or prasugrel in patients aged 70 years or older with non-ST-elevation acute coronary syndrome (POPular AGE): the randomised, open-label, non-inferiority trial. Lancet. 2020;395(10233):1374-81.

20. Ray KK, Nicholls SJ, Buhr KA, Ginsberg HN, Johansson JO, Kalantar-Zadeh K, et al. Effect of Apabetalone Added to Standard Therapy on Major Adverse Cardiovascular Events in Patients With Recent Acute Coronary Syndrome and Type 2 Diabetes: A Randomized Clinical Trial. JAMA. 2020;323(16):1565-73.

21. You J, Li H, Guo W, Li J, Gao L, Wang Y, et al. Platelet function testing guided antiplatelet therapy reduces cardiovascular events in Chinese patients with ST-segment elevation myocardial infarction undergoing percutaneous coronary intervention: The PATROL study. Catheter Cardiovasc Interv. 2020;95 Suppl 1:598-605.

22. Tardif JC, Kouz S, Waters DD, Bertrand OF, Diaz R, Maggioni AP, et al. Efficacy and Safety of Low-Dose Colchicine after Myocardial Infarction. N Engl J Med. 2019;381(26):2497-505.

23. Claassens DMF, Vos GJA, Bergmeijer TO, Hermanides RS, van 't Hof AWJ, van der Harst P, et al. A Genotype-Guided Strategy for Oral P2Y12 Inhibitors in Primary PCI. N Engl J Med. 2019;381(17):1621-31.

24. Schupke S, Neumann FJ, Menichelli M, Mayer K, Bernlochner I, Wohrle J, et al. Ticagrelor or Prasugrel in Patients with Acute Coronary Syndromes. N Engl J Med. 2019;381(16):1524-34.

25. De Luca G, Damen SA, Camaro C, Benit E, Verdoia M, Rasoul S, et al. Final results of the randomised evaluation of short-term dual antiplatelet therapy in patients with acute coronary syndrome treated with a new-generation stent (REDUCE trial). EuroIntervention. 2019;15(11):e990-e8.

26. Watanabe H, Ozasa N, Morimoto T, Shiomi H, Bingyuan B, Suwa S, et al. Long-term use of carvedilol in patients with ST-segment elevation myocardial infarction treated with primary percutaneous coronary intervention. PLoS ONE. 2018;13(8):e0199347.

27. Hahn JY, Song YB, Oh JH, Cho DK, Lee JB, Doh JH, et al. 6-month versus 12-month or longer dual antiplatelet therapy after percutaneous coronary intervention in patients with acute coronary syndrome (SMART-DATE): a randomised, open-label, non-inferiority trial. Lancet. 2018;391(10127):1274-84.

28. Notarangelo FM, Maglietta G, Bevilacqua P, Cereda M, Merlini PA, Villani GQ, et al. Pharmacogenomic Approach to Selecting Antiplatelet Therapy in Patients With Acute Coronary Syndromes: The PHARMCLO Trial. J Am Coll Cardiol. 2018;71(17):1869-77.

29. Sibbing D, Aradi D, Jacobshagen C, Gross L, Trenk D, Geisler T, et al. Guided de-escalation of antiplatelet treatment in patients with acute coronary syndrome undergoing percutaneous coronary intervention (TROPICAL-ACS): a randomised, open-label, multicentre trial. Lancet. 2017;390(10104):1747-57.

30. Cuisset T, Deharo P, Quilici J, Johnson TW, Deffarges S, Bassez C, et al. Benefit of switching dual antiplatelet therapy after acute coronary syndrome: the TOPIC (timing of platelet inhibition after acute coronary syndrome) randomized study. Eur Heart J. 2017;38(41):3070-8.

31. Hagiwara N, Kawada-Watanabe E, Koyanagi R, Arashi H, Yamaguchi J, Nakao K, et al. Low-density lipoprotein cholesterol targeting with pitavastatin + ezetimibe for patients with acute coronary syndrome and dyslipidaemia: the HIJ-PROPER study, a prospective, open-label, randomized trial. Eur Heart J. 2017;38(29):2264-76.

32. Liu Z, Hao H, Yin C, Chu Y, Li J, Xu D. Therapeutic effects of atorvastatin and ezetimibe compared with double-dose atorvastatin in very elderly patients with acute coronary syndrome. Oncotarget. 2017;8(25):41582-9.

33. O'Donoghue ML, Braunwald E, White HD, Lukas MA, Tarka E, Steg PG, et al. Effect of darapladib on major coronary events after an acute coronary syndrome: the SOLID-TIMI 52 randomized clinical trial. JAMA. 2014;312(10):1006-15.

34. White WB, Cannon CP, Heller SR, Nissen SE, Bergenstal RM, Bakris GL, et al. Alogliptin after acute coronary syndrome in patients with type 2 diabetes. N Engl J Med. 2013;369(14):1327-35.

35. Schwartz GG, Olsson AG, Abt M, Ballantyne CM, Barter PJ, Brumm J, et al. Effects of dalcetrapib in patients with a recent acute coronary syndrome. N Engl J Med. 2012;367(22):2089-99.

36. Hanash JA, Hansen BH, Hansen JF, Nielsen OW, Rasmussen A, Birket-Smith M. Cardiovascular safety of one-year escitalopram therapy in clinically nondepressed patients with acute coronary syndrome: results from the DEpression in patients with Coronary ARtery Disease (DECARD) trial. J Cardiovasc Pharmacol. 2012;60(4):397-405.

37. Mega JL, Braunwald E, Wiviott SD, Bassand JP, Bhatt DL, Bode C, et al. Rivaroxaban in patients with a recent acute coronary syndrome. N Engl J Med. 2012;366(1):9-19.

38. Wallentin L, Becker RC, Budaj A, Cannon CP, Emanuelsson H, Held C, et al. Ticagrelor versus clopidogrel in patients with acute coronary syndromes. N Engl J Med. 2009;361(11):1045-57.

39. Morrow DA, Scirica BM, Karwatowska-Prokopczuk E, Murphy SA, Budaj A, Varshavsky S, et al. Effects of ranolazine on recurrent cardiovascular events in patients with non-ST-elevation acute coronary syndromes: the MERLIN-TIMI 36 randomized trial. JAMA. 2007;297(16):1775-83.

40. Sakamoto T, Kojima S, Ogawa H, Shimomura H, Kimura K, Ogata Y, et al. Effects of early statin treatment on symptomatic heart failure and ischemic events after acute myocardial infarction in Japanese. Am J Cardiol. 2006;97(8):1165-71.

41. Cannon CP, Braunwald E, McCabe CH, Grayston JT, Muhlestein B, Giugliano RP, et al. Antibiotic treatment of Chlamydia pneumoniae after acute coronary syndrome. N Engl J Med. 2005;352(16):1646-54.

42. Ueshima K, Fukami K, Hiramori K, Hosoda S, Kishida H, Kato K, et al. Is angiotensin-converting enzyme inhibitor useful in a Japanese population for secondary prevention after acute myocardial infarction? A final report of the Japanese Acute Myocardial Infarction Prospective (JAMP) study. Am Heart J. 2004;148(2):e8.

43. Cannon CP, Braunwald E, McCabe CH, Rader DJ, Rouleau JL, Belder R, et al. Intensive versus moderate lipid lowering with statins after acute coronary syndromes. N Engl J Med. 2004;350(15):1495-504.

44. Liem AH, van Boven AJ, Veeger NJ, Withagen AJ, Robles de Medina RM, Tijssen JG, van Veldhuisen DJ. Efficacy of folic acid when added to statin therapy in patients with hypercholesterolemia following acute myocardial infarction: a randomised pilot trial. Int J Cardiol. 2004;93(2-3):175-9.

45. Herlitz J, Holm J, Peterson M, Karlson BW, Haglid Evander M, Erhardt L. Effect of fixed low-dose warfarin added to aspirin in the long term after acute myocardial infarction; the LoWASA Study. Eur Heart J. 2004;25(3):232-9.

46. Pfeffer MA, McMurray JJ, Velazquez EJ, Rouleau JL, Kober L, Maggioni AP, et al. Valsartan, captopril, or both in myocardial infarction complicated by heart failure, left ventricular dysfunction, or both. N Engl J Med. 2003;349(20):1893-906.

47. Pitt B, Remme W, Zannad F, Neaton J, Martinez F, Roniker B, et al. Eplerenone, a selective aldosterone blocker, in patients with left ventricular dysfunction after myocardial infarction. N Engl J Med. 2003;348(14):1309-21.

48. Hurlen M, Abdelnoor M, Smith P, Erikssen J, Arnesen H. Warfarin, aspirin, or both after myocardial infarction. N Engl J Med. 2002;347(13):969-74.

49. Torp-Pedersen C, Kober L, Ball S, Hall A, Brendorp B, Ottesen M, et al. The incomplete bucindolol evaluation in acute myocardial infarction Trial (BEAT). Eur J Heart Fail. 2002;4(4):495-9.

50. Nilsen DW, Albrektsen G, Landmark K, Moen S, Aarsland T, Woie L. Effects of a high-dose concentrate of n-3 fatty acids or corn oil introduced early after an acute myocardial infarction on serum triacylglycerol and HDL cholesterol. Am J Clin Nutr. 2001;74(1):50-6.

51. Huynh T, Theroux P, Bogaty P, Nasmith J, Solymoss S. Aspirin, warfarin, or the combination for secondary prevention of coronary events in patients with acute coronary syndromes and prior coronary artery bypass surgery. Circulation. 2001;103(25):3069-74.

52. Dargie HJ. Effect of carvedilol on outcome after myocardial infarction in patients with left-ventricular dysfunction: the CAPRICORN randomised trial. Lancet. 2001;357(9266):1385-90.

53. Kober L, Bloch Thomsen PE, Moller M, Torp-Pedersen C, Carlsen J, Sandoe E, et al. Effect of dofetilide in patients with recent myocardial infarction and left-ventricular dysfunction: a randomised trial. Lancet. 2000;356(9247):2052-8.

54. Ishikawa K, Kanamasa K, Hama J, Ogawa I, Takenaka T, Naito T, et al. Aspirin plus either dipyridamole or ticlopidine is effective in preventing recurrent myocardial infarction. Secondary Prevention Group. Jpn Circ J. 1997;61(1):38-45.

55. Kober L, Torp-Pedersen C, Carlsen JE, Bagger H, Eliasen P, Lyngborg K, et al. A clinical trial of the angiotensin-converting-enzyme inhibitor trandolapril in patients with left ventricular dysfunction after myocardial infarction. Trandolapril Cardiac Evaluation (TRACE) Study Group. N Engl J Med. 1995;333(25):1670-6.

56. Condorelli M, Chiariello M, Dagianti A, Penco M, Dalla Volta S, Pengo V, et al. IPO-V2: a prospective, multicenter, randomized, comparative clinical investigation of the effects of sulodexide in preventing cardiovascular accidents in the first year after acute myocardial infarction. J Am Coll Cardiol. 1994;23(1):27-34.

57. Galloe AM, Rasmussen HS, Jorgensen LN, Aurup P, Balslov S, Cintin C, et al. Influence of oral magnesium supplementation on cardiac events among survivors of an acute myocardial infarction. BMJ. 1993;307(6904):585-7.

58. Ceremuzynski L, Kleczar E, Krzeminska-Pakula M, Kuch J, Nartowicz E, Smielak-Korombel J, et al. Effect of amiodarone on mortality after myocardial infarction: a double-blind, placebo-controlled, pilot study. J Am Coll Cardiol. 1992;20(5):1056-62.

59. Cairns JA, Gent M, Singer J, Finnie KJ, Froggatt GM, Holder DA, et al. Aspirin, sulfinpyrazone, or both in unstable angina. Results of a Canadian multicenter trial. N Engl J Med. 1985;313(22):1369-75.

60. Julian DG, Prescott RJ, Jackson FS, Szekely P. Controlled trial of sotalol for one year after myocardial infarction. Lancet. 1982;1(8282):1142-7.

61. Peter T, Ross D, Duffield A, Luxton M, Harper R, Hunt D, Sloman G. Effect on survival after myocardial infarction of long-term treatment with phenytoin. Br Heart J. 1978;40(12):1356-60.

62. Wu HB, Tian HP, Wang XC, Bai SR, Li XN, Zhang LN, Du RP. Clinical efficacy of ticagrelor in patients undergoing emergency intervention for acute myocardial infarction and its impact on platelet aggregation rate. Am J Transl Res. 2018;10(7):2175-83.

63. Navarro-Lopez F, Cosin J, Marrugat J, Guindo J, De Luna AB. Comparison of the effects of amiodarone versus metoprolol on the frequency of ventricular arrhythmias and on mortality after acute myocardial infarction. AM J CARDIOL. 1993;72(17):1243-8.

64. Colivicchi F, Tubaro M, Mocini D, Genovesi Ebert A, Strano S, Melina G, et al. Full-dose atorvastatin versus conventional medical therapy after non-ST-elevation acute myocardial infarction in patients with advanced non-revascularisable coronary artery disease. Curr Med Res Opin. 2010;26(6):1277-84.

65. Tardif JC, Pfeffer MA, Kouz S, Koenig W, Maggioni AP, McMurray JJV, et al. Pharmacogenetics-guided dalcetrapib therapy after an acute coronary syndrome: The dal-GenE trial. Eur Heart J. 2022;43(39):3947-56.

66. Zhu HC, Li Y, Guan SY, Li J, Wang XZ, Jing QM, et al. Efficacy and safety of individually tailored antiplatelet therapy in patients with acute coronary syndrome after coronary stenting: A single center, randomized, feasibility study. J Geriatr Cardiol. 2015;12(1):23-9.

67. Tong DC, Quinn S, Nasis A, Hiew C, Roberts-Thomson P, Adams H, et al. Colchicine in Patients With Acute Coronary Syndrome The Australian COPS Randomized Clinical Trial. Circulation. 2020;142(20):1890-900.

68. Liem AH, Van Boven AJ, Veeger NJGM, Withagen AJ, Robles de Medina RM, Tijssens JGP, Van Veldhuisen DJ. Effect of fluvastatin on ischaemia following acute myocardial infarction: A randomized trial. Eur Heart J. 2002;23(24):1931-7.

69. Savonitto S, Ferri LA, Piatti L, Grosseto D, Piovaccari G, Morici N, et al. Comparison of reduced-dose prasugrel and standard-dose clopidogrel in elderly patients with acute coronary syndromes undergoing early percutaneous revascularization. Circulation. 2018;137(23):2435-45.

70. Pfeffer MA, Braunwald E, Moye LA, Basta L, Brown Jr EJ, Cuddy TE, et al. Effect of captopril on mortality and morbidity in patients with left ventricular dysfunction after myocardial infarction - Results of the survival and ventricular enlargement trial. NEW ENGL J MED. 1992;327(10):669-77.

71. Park S, Rha SW, Choi BG, Kim W, Choi WG, Lee SJ, et al. Efficacy and safety of cilostazol-based triple antiplatelet therapy compared with clopidogrel-based dual antiplatelet therapy in patients with acute ST-elevation myocardial infarction undergoing percutaneous coronary intervention: A multicenter, randomize. Am Heart J. 2023;265:11-21.

72. Liu Z, Xu Y, Hao H, Yin C, Xu J, Li J, et al. Efficacy of high intensity atorvastatin versus moderate intensity atorvastatin for acute coronary syndrome patients with diabetes mellitus. Int J Cardiol. 2016;222:22-6.

73. Mrdovic IB, Savic LZ, Perunicic JP, Asanin MR, Lasica RM, Jelena MM, et al. Randomized active-controlled study comparing effects of treatment with carvedilol versus metoprolol in patients with left ventricular dysfunction after acute myocardial infarction. Am Heart J. 2007;154(1):116-22.

74. Yasue H, Ogawa H, Tanaka H, Miyazaki S, Hattori R, Saito M, et al. Effects of aspirin and trapidil on cardiovascular events after acute myocardial infarction. Am J Cardiol. 1999;83(9):1308-13.

75. Chi H, Wang S, Chen J, Zhang J. Long-term effects of simvastatin on protection against atrial fibrillation in patients with acute myocardial infarction. J Geriatr Cardiol. 2007;4(3):144-7.

76. Nosaka K, Miyoshi T, Iwamoto M, Kajiya M, Okawa K, Tsukuda S, et al. Early initiation of eicosapentaenoic acid and statin treatment is associated with better clinical outcomes than statin alone in patients with acute coronary syndromes: 1-year outcoomes. European heart journal. 2016;37:1391.

77. Julian DG, Camm AJ, Frangin G, Janse MJ, Munoz A, Schwartz PJ, Simon P. Randomised trial of effect of amiodarone on mortality in patients with left-ventricular dysfunction after recent myocardial infarction: EMIAT. European Myocardial Infarct Amiodarone Trial Investigators. Lancet (london, england). 1997;349(9053):667-74.

78. Pfeffer MA, Claggett B, Lewis EF, Granger CB, Kober L, Maggioni AP, et al. Angiotensin Receptor-Neprilysin Inhibition in Acute Myocardial Infarction. N Engl J Med. 2021;385(20):1845-55.

79. Rauch B, Schiele R, Schneider S, Diller F, Victor N, Gohlke H, et al. OMEGA, a randomized, placebo-controlled trial to test the effect of highly purified omega-3 fatty acids on top of modern guideline-adjusted therapy after myocardial infarction. Circulation. 2010;122(21):2152-9.

80. Bonaa KH, Njolstad I, Ueland PM, Schirmer H, Tverdal A, Steigen T, et al. Homocysteine lowering and cardiovascular events after acute myocardial infarction. N Engl J Med. 2006;354(15):1578-88.

81. Anonymous. Effect of long-term oral anticoagulant treatment on mortality and cardiovascular morbidity after myocardial infarction. Anticoagulants in the Secondary Prevention of Events in Coronary Thrombosis (ASPECT) Research Group. Lancet. 1994;343(8896):499-503.

82. Jespersen CM. The effect of verapamil on major events in patients with impaired cardiac function recovering from acute myocardial infarction. The Danish Study Group on Verapamil in Myocardial Infarction. Eur Heart J. 1993;14(4):540-5.

83. Anonymous. Effect of ramipril on mortality and morbidity of survivors of acute myocardial infarction with clinical evidence of heart failure. The Acute Infarction Ramipril Efficacy (AIRE) Study Investigators. Lancet. 1993;342(8875):821-8.

84. Anonymous. European Infarction Study (E.I.S.). A secondary prevention study with slow release oxprenolol after myocardial infarction: morbidity and mortality. Eur Heart J. 1984;5(3):189-202.

85. Barber JM, Boyle DM, Chaturvedi NC, Singh N, Walsh MJ. Practolol in acute myocardial infarction. Acta Med Scand Suppl. 1976;587:213-9.

86. Anonymous. Improvement in prognosis of myocardial infarction by long-term beta-adrenoreceptor blockade using practolol. A multicentre international study. Br Med J. 1975;3(5986):735-40.

87. Breddin K, Loew D, Lechner K. Secondary prevention of myocardial infarction: A comparison of acetylsalicylic acid, placebo and phenprocoumon. HAEMOSTASIS. 1980;9(6):325-44.

88. Japanese b-B, Calcium Antagonists Myocardial Infarction I. Comparison of the effects of beta blockers and calcium antagonists on cardiovascular events after acute myocardial infarction in Japanese subjects. Am J Cardiol. 2004;93(8):969-73.

89. Study of the Effectiveness of Additional Reductions in C, Homocysteine Collaborative G, Armitage JM, Bowman L, Clarke RJ, Wallendszus K, et al. Effects of homocysteine-lowering with folic acid plus vitamin B12 vs placebo on mortality and major morbidity in myocardial infarction survivors: a randomized trial. JAMA. 2010;303(24):2486-94.

90. Burkart F, Pfisterer M, Kiowski W, Follath F, Burckhardt D. Effect of antiarrhythmic therapy on mortality in survivors of myocardial infarction with asymptomatic complex ventricular arrhythmias: Basel Antiarrhythmic Study of Infarct Survival (BASIS). J Am Coll Cardiol. 1990;16(7):1711-8.

91. Randomised double-blind trial of fixed low-dose warfarin with aspirin after myocardial infarction. The Lancet. 1997;350(9075):389-96.

92. PHENYTOIN AFTER RECOVERY FROM MYOCARDIAL INFARCTION: Controlled Trial in 568 Patients COLLABORATIVE GROUP. The Lancet. 1971;298(7733):1055-7.

93. Brie DM, Mornos C, Brie DA, Luca CT, Petrescu L, Boruga M. Potential role for pentoxifylline as an anti-inflammatory drug for patients with acute coronary syndrome. Exp Ther Med. 2022;23(6):378.

94. Multicenter Diltiazem Postinfarction Trial Research G. The effect of diltiazem on mortality and reinfarction after myocardial infarction. N Engl J Med. 1988;319(7):385-92.

95. Lincoff AM, Tardif JC, Schwartz GG, Nicholls SJ, Rydén L, Neal B, et al. Effect of aleglitazar on cardiovascular outcomes after acute coronary syndrome in patients with type 2 diabetes mellitus: the AleCardio randomized clinical trial. Jama. 2014;311(15):1515-25.

96. Anturane Reinfarction Trial Research G. Sulfinpyrazone in the prevention of cardiac death after myocardial infarction. The Anturane Reinfarction Trial. N Engl J Med. 1978;298(6):289-95.

97. Norwegian Multicenter Study G. Timolol-induced reduction in mortality and reinfarction in patients surviving acute myocardial infarction. N Engl J Med. 1981;304(14):801-7.

98. Butler J, Jones WS, Udell JA, Anker SD, Petrie MC, Harrington J, et al. Empagliflozin after Acute Myocardial Infarction. N Engl J Med. 2024;390(16):1455-66.

99. Gao C, Zhu B, Ouyang F, Wen S, Xu Y, Jia W, et al. Stepwise dual antiplatelet therapy de-escalation in patients after drug coated balloon angioplasty (REC-CAGEFREE II): multicentre, randomised, open label, assessor blind, non-inferiority trial. Bmj. 2025;388:e082945.

100. Jang Y, Park SD, Lee JP, Choi SH, Kong MG, Won YS, et al. One-month dual antiplatelet therapy followed by prasugrel monotherapy at a reduced dose: the 4D-ACS randomised trial. EuroIntervention. 2025;21(14):e796-e809.

101. Yndigegn T, Lindahl B, Mars K, Alfredsson J, Benatar J, Brandin L, et al. Beta-Blockers after Myocardial Infarction and Preserved Ejection Fraction. N Engl J Med. 2024;390(15):1372-81.

102. Li Y, Ren Y, Cheng L, Zhou X, Li W, Zhang L, et al. Efficacy of ivabradine in patients with poor heart rate control after beta-blocker use in acute myocardial infarction: a pragmatic randomized controlled trial. Front Cardiovasc Med. 2025;12:1560639.

|  |  | **Males** | | | | | **Females** | | | | | **REM males** | | | | **REM females** | | | |
| --- | --- | --- | --- | --- | --- | --- | --- | --- | --- | --- | --- | --- | --- | --- | --- | --- | --- | --- | --- |
|  |  | **Exp** | | **Ctrl** | |  | **Exp** | | **Ctrl** | |  |  |  |  |  |  |  |  |  |
| **Trial / First author** | **Year** | **N** | **EP** | **N** | **EP** | **EP (total)** | **N** | **EP** | **N** | **EP** | **EP (total)** | **Measure**  **^*)^ = calc** | **Estimate** | **UL** | **LL** | **Measure**  **^*)^ = calc** | **Estimate** | **UL** | **LL** |
| Secondary prevention of myocardial infarction | 1980 | 248 | 18 | 238 | 25 | 43 | 69 | 9 | 71 | 7 | 16 | OR* | 0.89 | 0.47 | 1.67 | OR* | 1.37 | 0.48 | 3.91 |
|  |  | 257 | 30 | 238 | 25 | 55 | 63 | 9 | 71 | 7 | 16 | OR* | 1.50 | 0.86 | 2.62 | OR* | 1.52 | 0.53 | 4.36 |
| CARS | 1997 | 1589 | x | 2586 | x | x | 439 | x | 800 | x | x | RR* | 1.01 | 0.86 | 1.20 | RR* | 1.07 | 0.82 | 1.41 |
|  |  | 2622 | x | 2586 | x | x | 758 | x | 800 | x | x | RR* | 0.99 | 0.77 | 1.17 | RR* | 0.93 | 0.67 | 1.30 |
| PLATO | 2009 | 6678 | 614 | 6658 | 739 | 1353 | 2655 | 297 | 2633 | 348 | 645 | HR | 0.85 | 0.76 | 0.95 | HR | 0.83 | 0.71 | 0.97 |
| APPRAISE-2 | 2011 | 2496 | x | 2518 | x | 362 | 1209 | x | 1169 | x | 210 | HR | 0.88 | 0.71 | 1.08 | HR | 1.08 | 0.83 | 1.42 |
| TRACER trial | 2012 | 4663 | x | 4649 | x | 1529 | 1810 | x | 1822 | x | 604 | HR | 0.90 | 0.81 | 0.99 | HR | 0.99 | 0.85 | 1.17 |
| TRILOGY ACS | 2012 | 2310 | 309 | 2333 | 387 | x | 1310 | 193 | 1290 | 191 | x | HR | 0.86 | 0.72 | 1.03 | HR | 1.02 | 0.80 | 1.29 |
| ATLAS ACS-2 TIMI 51 | 2012 | 7718 | 464 | 3882 | 270 | 734 | 2632 | 162 | 1294 | 106 | 268 | HR | 0.87 | 0.75 | 1.01 | HR | 0.77 | 0.60 | 0.99 |
| TROPICAL-ACS | 2017 | 1029 | x | 1023 | x | x | 275 | x | 283 | x | x | HR | 0.78 | 0.57 | 1.06 | HR | 0.92 | 0.53 | 1.62 |
| SMART-DATE | 2018 | 1016 | 48 | 1028 | 36 | 84 | 341 | 15 | 327 | 20 | 35 | HR | 1.37 | 0.89 | 2.11 | HR | 0.71 | 0.36 | 1.38 |
| Elderly ACS 2 | 2018 | 419 | x | 448 | x | 141 | 294 | x | 282 | x | 101 | HR | 0.99 | 0.71 | 1.38 | HR | 1.01 | 0.69 | 1.50 |
| POPular Genetics | 2019 | 925 | 44 | 937 | 51 | 95 | 317 | 19 | 309 | 22 | 41 | HR | 0.87 | 0.58 | 1.30 | HR | 0.84 | 0.46 | 1.56 |
| REDUCE-it | 2019 | 620 | 73 | 576 | 70 | 143 | 131 | 12 | 169 | 18 | 30 | HR | 0.96 | 0.70 | 1.34 | HR | 0.85 | 0.41 | 1.77 |
| ISAR-REACT 5 | 2019 | 1534 | 142 | 1528 | 98 |  | 478 | 42 | 478 | 39 | x | HR | 1.47 | 1.13 | 1.90 | HR | 1.10 | 0.71 | 1.70 |
| POPular AGE | 2020 | 313 | x | 325 | x | 186 | 187 | x | 177 | x | 114 | HR | 0.73 | 0.54 | 0.97 | HR | 1.02 | 0.71 | 1.48 |
| HOST-REDUCE-POLYTECH-ACS | 2020 | 1050 | 75 | 1037 | 99 | x | 120 | 7 | 131 | 17 | x | HR | 0.74 | 0.55 | 1.00 | HR | 0.43 | 0.18 | 1.04 |
| TALOS-AMI | 2021 | 1132 | 53 | 1111 | 87 | 140 | 217 | 6 | 237 | 17 | 23 | HR | 0.58 | 0.41 | 0.82 | HR | 0.39 | 0.16 | 0.98 |
| Shi et al. | 2021 | 147 | 3 | 79 | 8 | 11 | 54 | 3 | 21 | 2 | 5 | HR | 0.19 | 0.050 | 0.70 | HR | 0.57 | 0.10 | 3.43 |
| STOPDAPT-2 ACS | 2022 | 1631 | 51 | 1649 | 47 | 98 | 427 | 14 | 429 | 11 | 25 | HR | 1.10 | 0.74 | 1.64 | HR | 1.29 | 0.58 | 2.83 |
| T-PASS | 2024 | 1193 | 33 | 1181 | 56 | 89 | 233 | 7 | 243 | 17 | 24 | HR | 0.58 | 0.38 | 0.89 | HR | 0.42 | 0.18 | 1.02 |
| REC-CAGEFREE II | 2025 | 727 | 64 | 733 | 67 | 131 | 248 | 23 | 240 | 17 | 40 | HR | 0.97 | 0.69 | 1.36 | HR | 1.33 | 0.71 | 2.50 |
| 4D-ACS | 2025 | 269 | 12 | 273 | 24 | 36 | 59 | 3 | 55 | 4 | 7 | HR | 0.50 | 0.25 | 0.99 | HR | 0.71 | 0.16 | 3.18 |
| Nilsen et al. | 2001 | 115 | 31 | 123 | 30 | 61 | 35 | 11 | 27 | 6 | 17 | HR | 1.1 | 0.66 | 1.81 | HR | 1.62 | 0.60 | 4.28 |
| PROVE IT–TIMI 22 | 2004 | 1634 | 376 | 1617 | 424 | 800 | 465 | 94 | 446 | 120 | 214 | HR* | 0.87 | 0.74 | 0.99 | HR* | 0.76 | 0.57 | 0.99 |
| dal-OUTCOMES | 2012 | 6365 | x | 6436 | x | x | 1573 | x | 1497 | x | x | HR | 1.07 | 0.95 | 1.21 | HR | 0.92 | 0.72 | 1.16 |
| ALPS-AMI | 2014 | 205 | x | 205 | x | x | 48 | x | 50 | x | x | HR | 2.29 | 0.99 | 5.27 | HR | 1.04 | 0.74 | 1.47 |
| SOLID-TIMI 52 | 2014 | 4847 | 641 | 4853 | 682 | 1323 | 1657 | 262 | 1669 | 228 | 490 | HR | 0.94 | 0.84 | 1.05 | HR | 1.17 | 0.98 | 1.40 |
| IMPROVE-IT | 2015 | 6842 | x | 6886 | x | x | 2225 | x | 2191 | x | x | HR | 0.95 | 0.90 | 1.01 | HR | 0.89 | 0.79 | 0.99 |
| HIJ-PROPER | 2017 | 639 | 212 | 661 | 252 | 464 | 225 | 71 | 196 | 64 | 135 | HR | 0.86 | 0.72 | 1.04 | HR | 1.00 | 0.71 | 1.40 |
| dal-GenE | 2022 | 2348 | 225 | 2404 | 252 | 477 | 723 | 67 | 672 | 75 | 142 | HR | 0.90 | 0.75 | 1.08 | HR | 0.82 | 0.59 | 1.13 |
| REDUCE-AMI | 2024 | 1945 | 152 | 1944 | 147 | 299 | 563 | 47 | 568 | 61 | 108 | HR | 1.02 | 0.81 | 1.28 | HR | 0.80 | 0.55 | 1.17 |
| SAVE | 1992 | 929 | 191 | 912 | 234 | 425 | 186 | 37 | 204 | 41 | 241 | OR* | 0.75 | 0.60 | 0.93 | OR* | 0.99 | 0.60 | 1.62 |
| AIRE | 1993 | 734 | x | 727 | x | x | 270 | x | 255 | x | x | HR* | 0.77 | 0.62 | 0.97 | HR* | 0.71 | 0.52 | 0.97 |
| TRACE | 1995 | 627 | 203 | 621 | 256 | 549 | 249 | 101 | 252 | 113 | 214 | RR | 0.74 | 0.62 | 0.89 | RR | 0.90 | 0.69 | 1.18 |
| VALIANT | 2003 | 3365 | x | 3373 | x | x | 1544 | x | 1536 | x | x | HR* | 1.02 | 0.94 | 1.11 | HR* | 0.97 | 0.86 | 1.07 |
|  |  | 3395 | x | 3373 | x | x | 1490 | x | 1536 | x | x | HR* | 1.05 | 0.95 | 1.14 | HR* | 0.99 | 0.87 | 1.11 |
| EPHESUS | 2003 | 2380 | x | 2334 | x | x | 939 | x | 979 | x | x | RR* | 0.86 | 0.75 | 1.04 | RR* | 0.78 | 0.65 | 0.97 |
| PARADISE-MI | 2021 | 2167 | x | 2131 | x | 514 | 663 | x | 700 | x | 197 | HR | 0.84 | 0.71 | 1.00 | HR | 1.10 | 0.83 | 1.45 |
| MDPIT | 1988 | 994 | 119 | 977 | 127 | 246 | 238 | 24 | 257 | 36 | 60 | HR | 0.95 | 0.77 | 1.18 | HR | 0.71 | 0.46 | 1.08 |
| CAMIAT | 1997 | 500 | 13 | 489 | 25 | 38 | 106 | 2 | 107 | 6 | 8 | OR* | 0.50 | 0.25 | 0.98 | OR* | 0.32 | 0.06 | 1.64 |
| DIAMOND | 2000 | 542 | 157 | 569 | 172 | 329 | 207 | 73 | 192 | 71 | 144 | HR | 0.94 | 0.76 | 1.17 | HR | 0.92 | 0.66 | 1.28 |
| Cannon et al. | 2005 | 1638 | x | 1613 | x | x | 438 | x | 473 | x | x | HR* | 0.94 | 0.83 | 1.05 | HR* | 1.06 | 0.83 | 1.38 |
| Bonaa et al. | 2006 | 684 | x | 705 | x | x | 253 | x | 238 | x | x | RR | 1.23 | 0.96 | 1.57 | RR | 1.10 | 0.75 | 1.61 |
| MERLIN-TIMI 36 | 2007 | 2173 | 464 | 2096 | 460 | 510 | 1106 | 232 | 1185 | 293 | 525 | HR | 0.98 | 0.86 | 1.12 | HR | 0.83 | 0.70 | 0.99 |
| SEARCH | 2010 | 5006 | 1312 | 5006 | 1290 | x | 1027 | 225 | 1025 | 230 | x | RR* | 1.03 | 0.95 | 1.11 | RR* | 1.12 | 0.93 | 1.35 |
| EXAMINE | 2013 | 1828 | 197 | 1823 | 193 | 390 | 873 | 107 | 856 | 122 | 229 | HR | 1.02 | 0.84 | 1.24 | HR | 0.87 | 0.67 | 1.12 |
| COLCOT | 2019 | 1894 | 94 | 1942 | 135 | x | 472 | 37 | 437 | 35 | x | HR | 0.70 | 0.54 | 0.91 | HR | 0.99 | 0.63 | 1.58 |
| COPS | 2020 | 322 | x | 310 | x | x | 74 | x | 89 | x | x | HR | 0.60 | 0.33 | 1.10 | HR | 0.83 | 0.30 | 2.26 |
| BETonMACE | 2020 | 907 | 102 | 893 | 117 | 219 | 305 | 23 | 313 | 32 | 55 | HR | 0.84 | 0.64 | 1.10 | HR | 0.79 | 0.46 | 1.36 |
| EMPACT-MI | 2024 | 2448 | 176 | 2449 | 219 | 395 | 812 | 91 | 813 | 79 | 170 | HR | 0.81 | 0.66 | 0.99 | HR | 1.14 | 0.85 | 1.55 |

**Supplemental Table 2**. Sex -specific relative effect measures of the primary endpoint
**Abbreviations used:** Exp = experimental; Ctrl = control; N = numbers of patients (sample size); EP = endpoint; UL = upper limit; LL = lower limit;
